# Hepatic Topology of Glycosphingolipids in *Schistosoma mansoni*-Infected Hamsters

**DOI:** 10.1021/acs.analchem.3c05846

**Published:** 2024-04-09

**Authors:** David Luh, Sven Heiles, Martin Roderfeld, Christoph G. Grevelding, Elke Roeb, Bernhard Spengler

**Affiliations:** † Institute of Inorganic and Analytical Chemistry, Justus Liebig University Giessen, 35392 Giessen, Germany; ‡ Leibniz-Institut für Analytische Wissenschaften—ISAS—e.V., 44139 Dortmund, Germany; § Lipidomics, Faculty of Chemistry, University of Duisburg-Essen, 45141 Essen, Germany; ∥ Gastroenterology, Justus Liebig University Giessen, 35392Giessen, Germany; ⊥ Institute for Parasitology, Justus Liebig University Giessen, 35392 Giessen, Germany

## Abstract

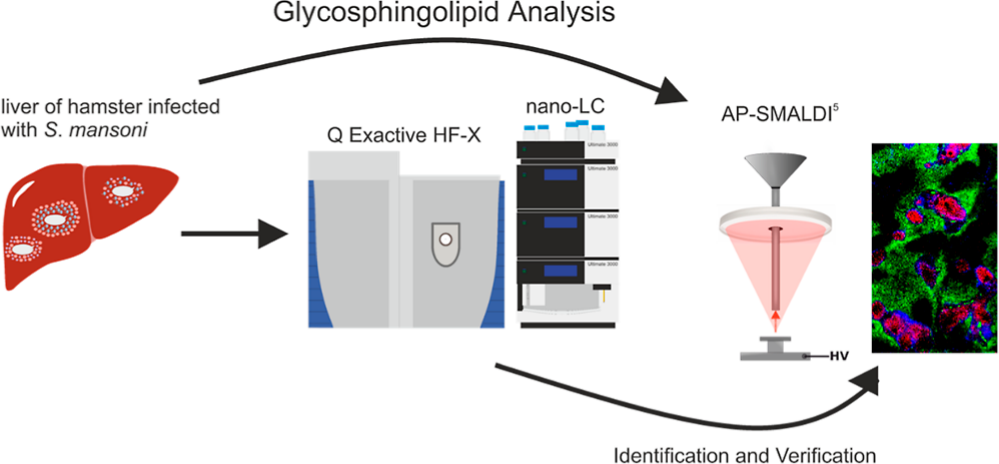

Schistosomiasis is a neglected tropical disease caused by worm parasites of the genus *Schistosoma*. Upon infection, parasite eggs can lodge inside of host organs like the liver. This leads to granuloma formation, which is the main cause of the pathology of schistosomiasis. To better understand the different levels of host–pathogen interaction and pathology, our study focused on the characterization of glycosphingolipids (GSLs). For this purpose, GSLs in livers of infected and noninfected hamsters were studied by combining high-spatial-resolution atmospheric-pressure scanning microprobe matrix-assisted laser desorption/ionization mass spectrometry imaging (AP-SMALDI MSI) with nanoscale hydrophilic interaction liquid chromatography tandem mass spectrometry (nano-HILIC MS/MS). Nano-HILIC MS/MS revealed 60 GSL species with a distinct saccharide and ceramide composition. AP-SMALDI MSI measurements were conducted in positive- and negative-ion mode for the visualization of neutral and acidic GSLs. Based on nano-HILIC MS/MS results, we discovered no downregulated but 50 significantly upregulated GSLs in liver samples of infected hamsters. AP-SMALDI MSI showed that 44 of these GSL species were associated with the granulomas in the liver tissue. Our findings suggest an important role of GSLs during granuloma formation.

## Introduction

Glycomics is an emerging field within omics-technologies, dealing with the structural and functional elucidation of N- and O-glycans, glycoproteins, or glycosphingolipids (GSLs). These molecular classes are involved in cellular communication as epitopes of pathogen recognition or play roles during immune response.^1^ Understanding the fundamental roles of these molecules is helpful to monitor metabolic processes or to facilitate drug and vaccine development.^2^ For these purposes, it is beneficial to analyze the spatial distribution of the analytes. A powerful molecular imaging technique is matrix-assisted laser desorption/ionization (MALDI) mass spectrometry imaging (MSI).^3,4^ In an MSI experiment, the distribution of several hundred molecular species can be resolved. This is a major advantage over other imaging methods such as immunohistochemistry (IHC). Here, individual staining is required for each antibody, and the total number of stainings within one experiment is limited to a few antibodies. However, the results of IHC can validate the MSI results and link them to specific cells or proteins, as shown in the work of Bien et al.^5^

A challenge for MSI in some cases is the low ionization efficiency for some compounds, which can result in low signal intensities, especially in high-spatial-resolution MALDI MSI.^6^ For high-resolution MALDI MSI, atmospheric-pressure scanning microprobe matrix-assisted laser desorption/ionization mass spectrometry imaging (AP-SMALDI MSI) with a spatial resolution of 1.4 μm has recently been developed.^7^

Successful AP-SMALDI MSI application was used for phospholipid analysis of livers of hamsters infected by *Schistosoma mansoni*.^8^ Besides other Schistosoma species, *S. mansoni* is responsible for schistosomiasis, an infectious disease classified as one of the neglected tropical diseases by the World Health Organization. Schistosomiasis affects over 200 million people worldwide and is mostly distributed in tropical and subtropical areas. The pathology of schistosomiasis can be divided into an acute and a chronic phase.^9^ Here, the chronic phase is more severe and can potentially lead to death.^10^ The morbidity during this stage is caused by eggs trapped inside organs, like the liver and spleen. Granuloma formation around eggs is the typical host response and can lead to chronic inflammation with excessive wound healing, leading to hepatic fibrosis.^11^*S. mansoni* eggs are even able to mobilize, incorporate, and store host lipids, thereby provoking hepatic exhaustion of neutral lipids and glycogen.^12^ To understand the host–parasite interaction, it is necessary to identify and characterize the molecules involved in signal transduction followed by the immune response. To the best of our knowledge, no studies have yet focused on the interaction between host and parasite eggs, leading to granuloma formation and resulting GSL responses. This, however, can be important because GSLs are involved in the immune responses of the host. GSLs, known to be crucial for signal transduction and membrane organization,^13^ are involved in forming microdomains essential for signal transduction in activated immune cells.^14^ Furthermore, they can directly regulate immune receptors, with GM3 as a regulator for the inhibition of insulin-induced signaling as one of the best understood examples.^15,16^ While some examples for GSL-specific roles are known, there is a general lack of analytical methods to study GSL profiles globally and locally.^17^

A promising approach to overcome these shortcomings is the combination of nanoscale hydrophilic interaction liquid chromatography tandem mass spectrometry (nano-HILIC MS/MS) with AP-SMALDI MSI to structurally characterize GSLs and to visualize their distributions in tissue sections.^3,4^ With this setup, we profiled hepatic GSLs in *S. mansoni*-infected hamsters. We analyzed, as controls, the livers of hamsters infected by only female worms (no egg production) or noninfected. We used nano-HILIC MS/MS for the detection of neutral and acidic GSLs to curate a GSL database.^18^ Statistic evaluation of nano-HILIC MS/MS data revealed significant differences between infected and noninfected samples. Subsequently, optimized AP-SMALDI MSI provided information about the topography of the GSL species in infected tissue. Semiquantitative evaluation of AP-SMALDI MSI data was found to be in line with nano-HILIC MS/MS results, providing evidence that AP-SMALDI MSI allows one to locally quantify GSLs. The abundance changes of GSL species during granuloma formation indicate a potential connection between GSLs and immune cell differentiation. Furthermore, AP-SMALDI MSI measurements of granulomas with 3 μm step size enabled us to resolve ultrafine structures.

## Experimental Section

### Chemicals

Acetonitrile, methanol, and water (HiPerSolv) were purchased from VWR International GmbH (Darmstadt, Germany). Chloroform (Rotipuran) was purchased from Carl Roth GmbH + Co. KG (Karlsruhe, Germany). 2,5-Dihydroxybenzoic acid (DHB), ethanol, glacial acetic acid, and trifluoroacetic acid were purchased from Merck (Darmstadt, Germany). Hematoxylin, eosin Y, Eukitt, and α-cyano-4-hydroxycinnamic acid (CHCA) were purchased from Sigma-Aldrich (Darmstadt, Germany). 1,5-Diaminonapthalene (DAN) was purchased from Acros Organics (Geel, Belgium). 2,5-Dihydroxyacetophenone (DHAP) and ammonium acetate were purchased from Alfa Aesar (Kandel, Germany). 9-Aminoacridine (9-AA) was purchased from TCI (Eschborn, Germany).

### Tissue and Sample Preparation

All animal experiments were approved by the Regierungspraesidium Giessen (V54-19 c 20/15 c GI 18/10 140 Nr. A26/2018) and performed in accordance with the European Convention for the Protection of Vertebrate Animals used for experimental and other scientific purposes (ETS no. 123; revised Appendix A). Tissue samples were obtained as described elsewhere.^8^ Briefly, three different groups of hamster livers were used. Hamsters infected with both sexes of *S. mansoni* cercariae (bs-infected), hamsters infected with only one sex of *S. mansoni* cercariae (ss-infected), and noninfected hamsters.^19−21^ For each group, three randomly chosen biological replicates were used throughout the study.

### AP-SMALDI MSI Sample Preparation

For AP-SMALDI MSI measurements, fresh frozen hamster livers were sectioned with a cryotome (Thermo Scientific Microm HM 525 Cryostat) and thaw mounted onto a microscopic slide. 20 μm thick sections were used for AP-SMALDI MSI measurements. Sections were stored at −80 °C until further use. During thawing, tissue slides were placed in a desiccator for 20 min followed by matrix application. The matrices 9-AA, DAN, DHB, and CHCA were applied by pneumatic spraying (SMALDIPrep, TransMIT GmbH, Giessen, Germany) with parameters listed in Table S1. For DHAP matrix application, sublimation experiments were performed with a home-built sublimation setup (Figure S1). Sublimation parameters are given in Table S1.

### Nano-HILIC Sample Preparation

Hamster liver homogenates were extracted without an exogenous standard, followed by saponification and SPE purification, to obtain GSL extracts for subsequent nano-HILIC MS/MS experiments. More detailed experimental procedures are included in Supplementary Protocol 1.

### AP-SMALDI MSI Experiments and Data Analysis

Measurements with 10 and 15 μm step size were performed on an AP-SMALDI^5^ AF ion source (TransMIT GmbH, Giessen, Germany)^22^ coupled to an orbital trapping mass spectrometer [Thermo Scientific Q Exactive HF, Thermo Fisher Scientific (Bremen) GmbH, Germany] with a mass resolution of 240,000 at *m*/*z* 200. The measurements with a 3 μm step size were performed using an ultrahigh-resolution prototype AP-SMALDI ion source (TransMIT GmbH)^7^ coupled to an orbital trapping mass spectrometer [Thermo Scientific Q Exactive, Thermo Fisher Scientific (Bremen) GmbH] with a mass resolution of 140,000 at *m*/*z* 200. More details are included in Supplementary Note 1.

The matrix DHAP was compared to DHB and CHCA in positive-ion mode for neutral GSLs. In negative-ion mode, DHAP was compared to 9-AA and DAN for acidic GSLs. Matrix evaluation was carried out by performing AP-SMALDI MSI measurements of consecutive mouse brain tissue sections on the same day. For each measurement, a region of interest (ROI) was generated with Mirion.^23^ The ROI represents the distribution patterns of GSL compounds used for comparison. Expected distribution patterns are known from previous studies.^24,25^ Signal intensities per pixel were calculated as the sum of the intensities of a GSL compound divided by the number of pixels in the ROI.

Raw-files were recalibrated with ReCal Offline. Ion-images were generated with Mirion. The brightness of the images was adjusted to provide better visualization. Statistical comparison of livers of *S. mansoni* bs-infected, ss-infected, and noninfected hamsters was carried out based on AP-SMALDI MSI results. For the bs-infected samples, two ROIs were defined, one including granulomas and eggs and one as a control without granulomatous tissue and without eggs. Figure S2f–h shows the ROIs for one biological replicate. In total, 2500 spectra for each biological replicate were statistically evaluated. Signal intensities of the evaluated compounds were summed up and divided by the sum of the total ion counts per pixel of all 2500 spectra in the ROI to obtain the mean intensities per pixel. Histograms were generated with Excel. Error bars represent the standard error. Statistical evaluation was performed with Perseus, with more details in Supplementary Note 2. Data underlying this study are openly available in the Metaspace database at https://metaspace2020.eu/project/Luh-GSL_in_liver upon publication.

### Nano-HILIC Experiments and Data Analysis

Nano-HILIC MS/MS measurements were performed with an UltiMate 3000 RSLCnano System (Thermo Fisher Scientific, Dreieich, Germany) equipped with an Accucore 150 amide-HILIC column (0.075 mm × 150 mm) coupled to an orbital trapping mass spectrometer [Thermo Scientific Q Exactive HF-X, Thermo Fisher Scientific (Bremen) GmbH]. The method was adapted from Bindila et al.^26^ with some modifications. More experimental details are included in Supplementary Note 3 and Tables S2 and S3.

Xcalibur was used to generate mass spectra and extract ion chromatograms. A GSL database was generated manually, and subsequently, nano-HILIC MS/MS data were processed with MZMine 2.33.^27,28^ For statistical analysis, Perseus^29^ was used, with details in Supplementary Note 4. Histograms were generated in Excel.

### Data Processing

All of the graphics and mass spectra shown were processed with CorelDraw.

### Nomenclature

To describe lipids and especially GSLs, the shorthand nomenclature of LIPIDMAPS^30,31^ and the nomenclature for glycans^32^ are used in this article. Simple hexoses, like glucose, galactose, or mannose, are abbreviated as Hex. Hexosamines are abbreviated HexNac and fucose as Fuc. The acidic saccharides *N*-acetylneuraminic acid and *N*-glycolylneuraminic acid are abbreviated as NeuAc and NeuGc, respectively. For example, a GSL with one *N*-acetylneuraminic acid and two hexoses and a ceramide with a D-erythro-sphingosine and a hexadecanoic acid is abbreviated as NeuAcHex_2_Cer 18:1;O2/16:0. The structure of monosaccharide units used in this article and the fragment ion nomenclature of GSLs after Domon and Costello and Merrill et al. are shown in Figure S5.^33,34^

## Results and Discussion

### *S. mansoni* Infection Raised the Hepatic Amount of Distinct GSLs

**Figure 1:**
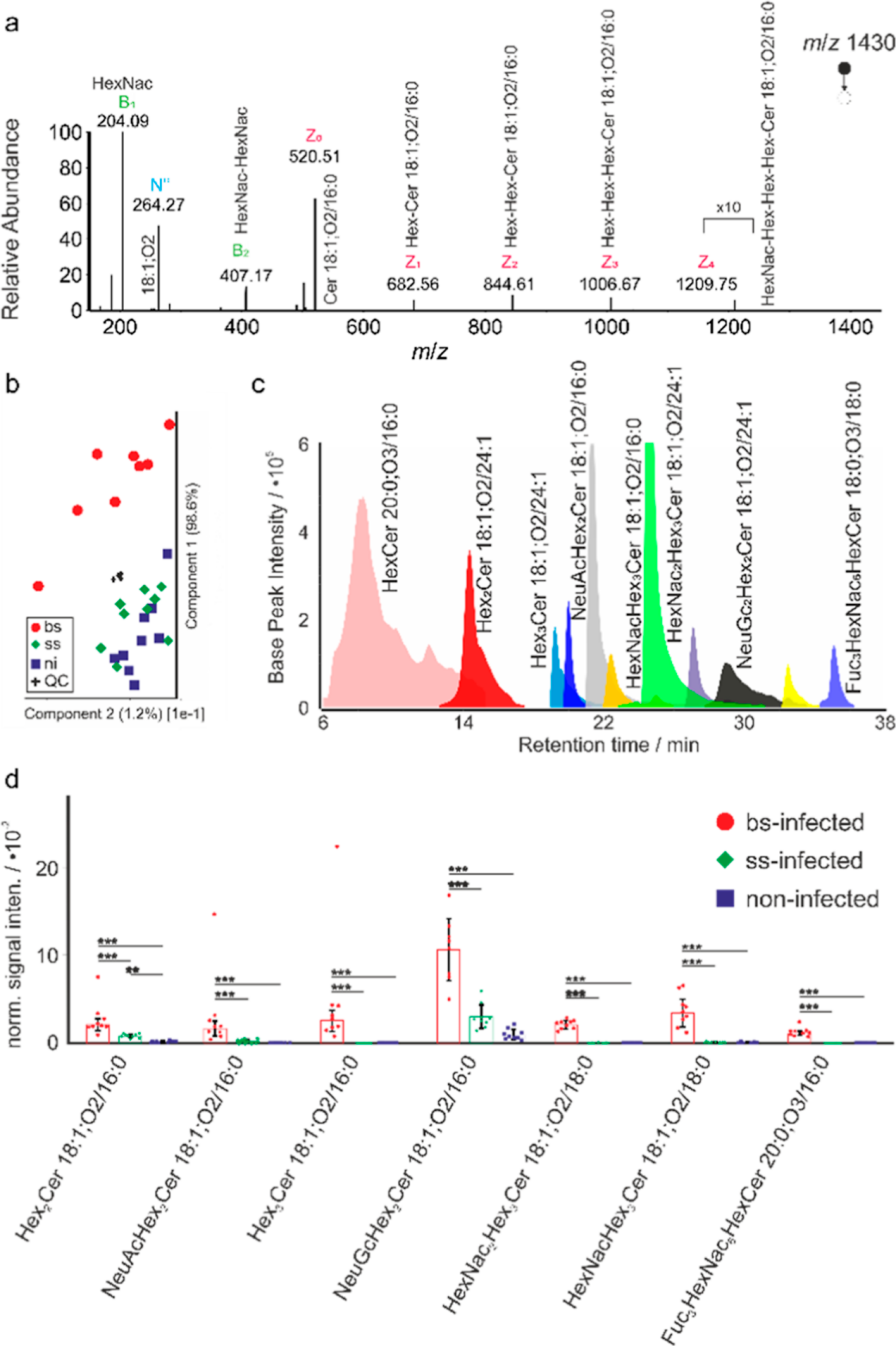
Nano-HILIC MS/MS analysis for GSL profiling. (a) Tandem mass spectrum for a singly charged precursor ion at *m*/*z* 1430, assigned as HexNac_2_Hex_3_Cer 18:1;O2/16:0, based on headgroup and backbone fragment ions. (b) Principal component analysis of nano-HILIC MS/MS data in positive-ion mode with “●” for bs-infected, “◊” for ss-infected, “□” for noninfected hamster, and “+” for quality control samples. (c) Extracted ion chromatogram (EIC) for GSLs from the liver of bs-infected hamsters. (d) Histograms for GSL species based on nano-HILIC MS/MS data. Black lines above two bars indicate the difference between the two corresponding samples, with “***” representing a significant difference with *p* < 0.001 and “**” with *p* < 0.01, respectively. Error bars show the standard error.

In order to characterize and quantify as many GSLs as possible, nano-HILIC MS/MS measurements with GSL extracts from the livers of hamsters infected with *S. mansoni* were performed. For these experiments, the main parameter for the separation of GSLs is their saccharide headgroup, with a representative chromatogram in Figure 1c. In total, we identified 60 molecularly different GSLs by ESI-MS/MS. For all 60 compounds, the ceramide backbone was determined, and for 47 compounds, the composition and sequence of the saccharide headgroup were assigned. A detailed list of the assigned GSL species is included in the Supporting Information. For acidic GSLs with more than three monosaccharide units (above 1400 Da), doubly charged ions were of higher signal intensities than singly charged GSLs, whereas for neutral GSLs, doubly charged ions above 1600 Da were more abundant than singly charged ions.

GSL ions were fragmented in positive- and negative-ion mode to resolve the saccharide headgroup composition and chemical makeup of the ceramide backbone. An example is shown in Figure 1a for a precursor ion at *m*/*z* 1430.8468. The Z-fragments (see Figure S5) at *m*/*z* 1209.75 and *m*/*z* 1006.67 correspond to neutral losses of hexosamine units. The next three Z-fragments at *m*/*z* 884.61, *m*/*z* 682.56, and *m*/*z* 520.51 correspond to consecutive neutral losses of hexose moieties. The complete series of Z-fragments allowed the saccharide sequence. Together with the sphingoid-base-specific *N*″-fragment, the structure of the GSL has been identified. The same feature is shown in Figure S6 for the negative-ion mode. Annotations for all other GSLs derived from MS^2^ results are summarized in the Supporting Information.

By conducting the experiments, we created a database. This was necessary because databases such as LIPIDMAPS are incomplete with respect to GSL compounds. This especially applies to parasite-specific GSLs.

The power of high resolution and high mass accuracy is apparent when the precursor intensity for MS^2^ experiments is low, resulting in an incomplete series of fragments for GSL annotation. Based on the accurate mass and the retention time, a reasonable annotation of an unknown GSL is still possible. Also, for compounds like Fuc_3_HexNac_7_HexCer 18:0;O3/18:0 with limited sequence coverage, a literature comparison can verify the annotation.^35^ Nanoscale liquid chromatography in comparison to normal liquid chromatography has the advantage of a higher sensitivity and improved dynamic range,^36^ beneficial for GSLs of low abundance. Currently, a bottleneck is the manual analysis of the nano-HILIC MS/MS data of GSLs. In order to investigate a potential link of GSL in granuloma formation upon *S. mansoni* infection, we compared the relative signal intensities of GSLs in the livers of noninfected, ss-infected, and bs-infected hamsters. For each group, nano-HILIC MS/MS measurements were conducted for three biological replicates, each measured as three technical replicates. The resulting data were first analyzed by principal component analysis (PCA) (Figure 1b). Separation of the groups was achieved based on GSL identities and signal intensities, indicating alterations of GSLs upon *S. mansoni* infection. Analysis of PCA loadings revealed that GSL species significantly contributed to the separation of the different groups. These GSL species and their abundance were statistically analyzed (Figure 1d). All identified GSLs that showed a change in abundance upon infection were upregulated. Downregulation was not observed. For example, Fuc_3_HexNac_6_HexCer 20:0;O3/16:0, which is an *S. mansoni*-egg-specific GSL, consequently represents a significant difference between bs-infected hamsters and ss-infected hamsters as well as controls (*p* < 0.001).

Other GSLs like HexNac_2_Hex_3_Cer 18:1;O2/18:0 or NeuAcHex_2_Cer 18:1;O2/16:0 were also detected in the liver of ss-infected hamsters but were found to be significantly increased (*p* < 0.001) in intensity upon bs-infection. Some compounds such as Hex_2_Cer 18:1;O2/16:0 showed significant upregulation not only in bs-infected animals (*p* < 0.001) but also in ss-infected animals (*p* < 0.01) compared to that observed in noninfected controls. Several compounds, including NeuGcHex_2_Cer 18:1;O2/16:0, were detected in all samples but with a significant increase in abundance (*p* < 0.001) in bs-infected individuals.

GSLs are known to be involved in immune response, during which several types of immune cells are recruited, including B-and T cells, eosinophils, and macrophages,^11,37,38^ which form granulomas around *Schistosoma manonsi* eggs. Therefore, the upregulation of GSLs in bs-infected hamsters compared to that in ss- and noninfected controls was expected. Unexpected were the different results for GSL species in ss-infected and noninfected animals. The nano-HILIC MS/MS data revealed six significantly upregulated GSLs in the ss group compared to noninfected controls. Although ss-infected hamsters are not expected to show egg deposition, earlier reports had shown that unpaired worms can induce immune responses.^39−41^ One possible mechanism is that up to 85% of unpaired female worms and 65% of unpaired male worms are found in the mouse liver after infection, which results in an accumulation of inflammatory cells in blood vessels and hepatic tissue.^39^ Additionally, worms fed on the host’s blood and regurgitated the digesta into the host’s blood circle. This especially leads to bacteria and hemosiderin deposition in the liver, which results in inflammatory infiltrates.^41^ Also, the accidental secretion of nonfertilized egg-like structures by unpaired female worms was reported to cause a host immune response.^40^

Overall, the nano-HILIC MS/MS data revealed that GSL identities and abundances were significantly altered upon *S. mansoni* infection. Next, we studied the local changes in GSLs via AP-SMALDI MSI to identify the histological features that may be associated with altered GSL profiles. Therefore, we developed a dedicated AP-SMALDI MSI workflow.

### DHAP Is a Suitable Matrix for GSL Analysis by AP-SMALDI MSI

Matrices such as DHB, CHCA, 9-AA, and DAN have been used for AP-SMALDI MSI of lipids. For GSLs, DHAP and its derivatives are known to serve as potent AP-SMALDI matrices.^42,43^ The performance of these matrices for efficiently ionizing GSLs was compared in experiments using mouse brain tissue sections. The results are summarized in Table S4. Comparison of AP-SMALDI MSI data of four HexCer-compounds acquired in positive-ion mode showed a 1.2- to 1.5-fold enhancement of signal intensities for DHAP compared to that for DHB and a fold change of 2.2 to 3.5 when compared to that for CHCA. In negative-ion mode, GSL signal intensities (NeuAcHexNacHex_3_Cer 36:1;O2 and NeuAcHexNacHex_2_Cer 36:1;O2) were increased 2.1- to 2.3-fold for DHAP compared to that for 9-AA and 3.9- to 6.6-fold compared to that for DAN. Additionally, a comparison on other lipid classes was performed showing similar annotation numbers for the different matrices, with more details in Supplementary Note 5. Overall, the results indicate that DHAP is best suited among the tested matrices for GSL analysis by AP-SMALDI MSI. Consequently, DHAP was used in all following AP-SMALDI MSI experiments.

### Distinct Neutral GSLs Were Enriched in Hepatic Granuloma of *S. mansoni*-Infected Hamsters

**Figure 2:**
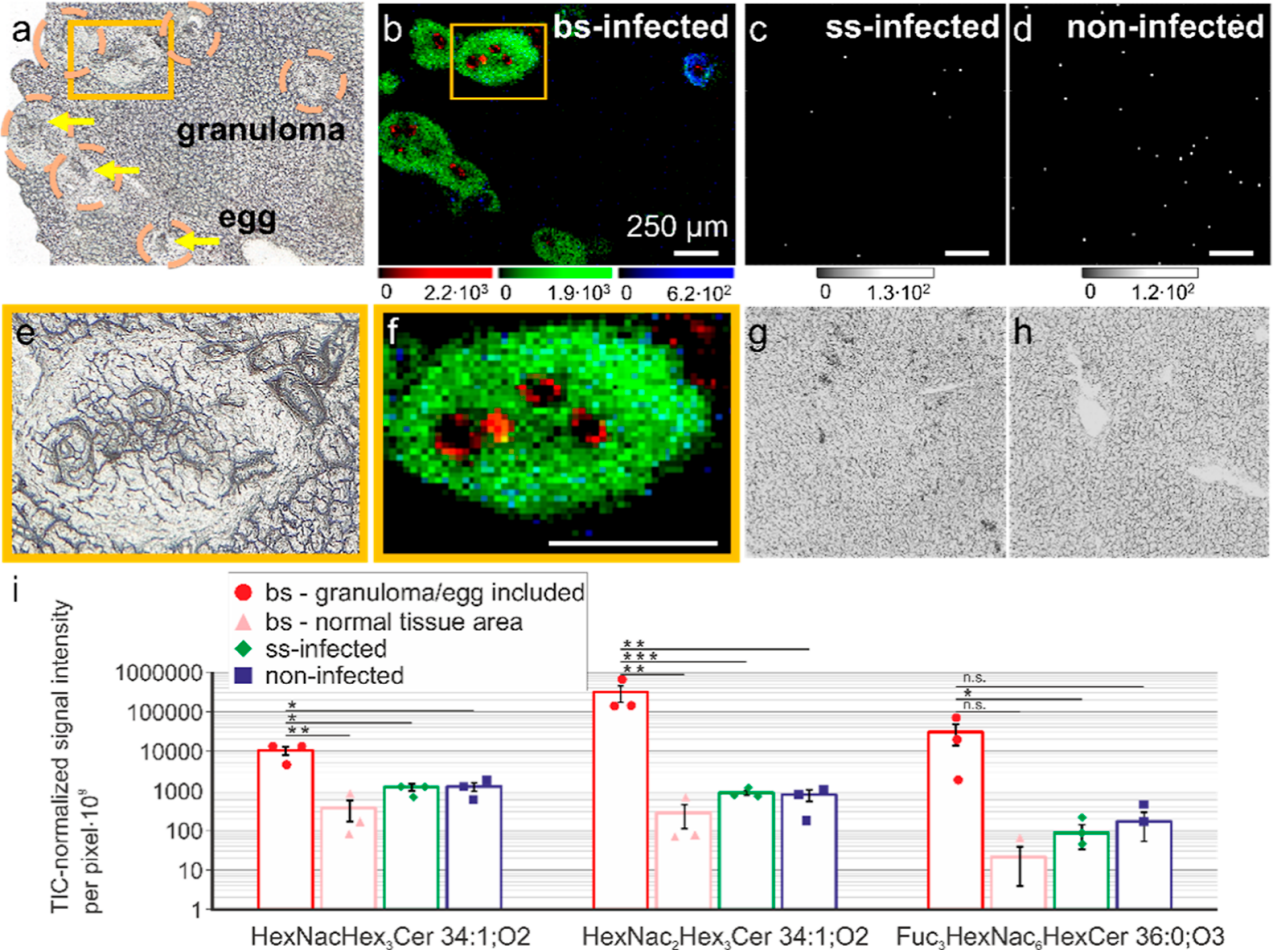
AP-SMALDI analysis of neutral GSLs. (a) Microscopic image of an *S. mansoni*-liver tissue section of bs-infected hamster, with yellow arrows exemplarily pointing at *S. mansoni* eggs and orange-dotted circles highlighting granulomas. (b) RGB image corresponding to the microscopic image in (a), showing Fuc_3_HexNac_6_HexCer 20:0;O3/16:0 ([M + K]^+^, at *m*/*z* 2442.2211) in red, HexNac_2_Hex_3_Cer 18:1;O2/16:0 ([M + K]^+^, at *m*/*z* 1468.7919) in green, and HexNacHex_3_Cer 18:1;O2/16:0 ([M + K]^+^, at *m*/*z* 1265.7134) in blue. Magnifications of parts (a,b) are shown in parts (e,f). (c) Ion image of a ss-infected hamster liver tissue section showing *m*/*z* 1468.7939 with the corresponding microscopic image (g). (d) Ion image of a noninfected hamster showing *m*/*z* 1468.7946 with the corresponding microscopic image (h). All scale bars are 250 μm. (i) Semiquantitative evaluation of ion images of Fuc_3_HexNac_6_HexCer 20:0;O3/16:0, HexNac_2_Hex_3_Cer 18:1;O2/16:0, and HexNacHex_3_Cer 18:1;O2/16:0, with a 50 × 50 pixel ROI showing the intensity per pixel for *n* = 3 with standard error as error bars. Red—bs-infected sample ROI with granuloma included, pink—bs-infected samples without granuloma included, green—ss-infected sample, and blue—noninfected sample. Black lines centered above two bars indicate the difference between the two corresponding ROIs, with “***” representing a significant difference with *p* < 0.001, “**” with *p* < 0.01, and “*” with *p* < 0.05. “n.s.” indicates a nonsignificant difference. Error bars show the standard error.

We employed our optimized AP-SMALDI MSI protocol to match the histological features with *S. mansoni* infection-specific GSLs, identified by nano-HILIC MS/MS. Liver tissue sections of bs-infected (*n* = 3), ss-infected (*n* = 3), and noninfected hamsters (*n* = 3) were measured. Representative images and results are shown in Figure 2. Data obtained in positive-ion mode were used to visualize neutral GSLs. A total of 25 ion images assigned to neutral GSL species, representing nine different saccharide compositions, were obtained from livers of bs-infected hamsters. Annotations were based on our nano-HILIC MS/MS database. Compounds were detected either in the granulomas (HexCer, Hex_2_Cer, Hex_3_Cer, HexNacHex_3_Cer, and HexNac_2_Hex_3_Cer) or in the *S. mansoni* eggs (HexCer, Fuc_3_HexNac_6_HexCer, Fuc_2_HexNac_6_HexCer, Fuc_3_HexNac_5_HexCer, and Fuc_3_HexNac_7_HexCer). For the ceramide composition, we observed mainly sphingosine 18:1;O2 for GSLs distributed in the hepatic tissue, with C16:0, C24:0, and C24:1 as the most prominent fatty acids. For egg-specific GSL, we found phytosphingosines as the dominant sphingoid base.

Egg-specific saccharide- and ceramide moieties of GSLs were investigated separately in a previous bulk analysis.^35^ We compared these results with our data for egg-specific GSLs obtained by AP-SMALDI MSI. As an example, the GSL Fuc_3_HexNac_6_HexCer 20:0;O3/16:0 at *m*/*z* 2442.2230 is shown in Figure 2b in red. The GSLs associated with the outer surface of the egg are in line with the previously reported data on saccharide and ceramide moieties of *S. mansoni* egg-specific GSLs.^35^ We were able to detect 5 out of the 17 previously reported saccharide compositions, with each composition appearing in one or several different complex GSL species. Interestingly, we only detected GSL compounds with a maximum number of three fucose moieties, different from up to eight fucose moieties of GSLs reported in the literature.^35^ Further studies are necessary to elucidate the reason for this discrepancy. Because our AP-SMALDI MSI and nano-HILIC MS data consistently indicated these five saccharide compounds, we assume biological variability between our sample material and that investigated in the previous study.

Besides the egg-specific GSLs, the observed distributions of hepatic GSLs are also in line with histological features visible in the corresponding optical image (Figure 2a). Examples for granuloma-specific GSL distributions are shown in Figure 2b, with HexNac_2_Hex_3_Cer 18:1;O2/16:0 as well as HexNacHex_3_Cer 18:1;O2/16:0 associated with granulomas. This indicates that specific GSLs are expressed within *S. mansoni* eggs and granulomas. To test this assumption, livers of ss- and noninfected hamsters were measured. The mass spectrometric images of HexNac_2_Hex_3_Cer 18:1;O2/16:0 in the liver sections of ss- and noninfected hamsters are shown in Figure 2c,d. Corresponding optical images are shown in Figure 2g,h. The ion-images indicated no accumulation of this GSL species in the tissues for either sample type. The same was found for the other granuloma-specific GSLs. Even though most granulomas could be associated with elevated signal intensities of HexNac_2_Hex_3_Cer 18:1;O2/16:0, some granulomas, relatively small in size, showed increased signal intensities of HexNacHex_3_Cer compared to those of HexNac_2_Hex_3_Cer (Figure 2b). Here, H&E staining also indicated differences between granulomas (Figure S7). The granuloma highlighted by a red-dotted circle shows a homogeneous distribution of HexNac_2_Hex_3_Cer. The granuloma highlighted by an orange-dotted circle shows a homogeneous distribution of HexNacHex_3_Cer and appears more purple, indicating a different cellular composition. This might indicate that different granuloma growth states are characterized by specific GSL species. For example, HexNacHex_3_Cer 18:1;O2/16:0 at *m*/*z* 1265.7135 in blue in Figure 2b was found to be highly abundant in only one granuloma in this tissue section and was found to accumulate only slightly at the borders of larger granulomas (magnified ion-image in Figure S2i). The same distribution as that for HexNacHex_3_Cer 18:1;O2/16:0 was observed for HexCer 18:1;O2/16:0, Hex_2_Cer 18:1;O2/16:0 and Hex_3_Cer 18:1;O2/16:0 (Figure S2j–l).

Granuloma formation is induced by the immune response and results in subsequent recruitment of immune cells in *S. mansoni* infections, allowing the observation of different stages of granuloma formation within the same tissue sample. This is also influenced by the time when an egg is trapped in the liver tissue because schistosomes constantly produce eggs in a host over many years.^11,44^ Against this background, we suggest that the signals specifically found in smaller granulomas, namely, Hex_2_Cer 18:1;O2/16:0, Hex_3_Cer 18:1;O2/16:0, and HexNacHex_3_Cer 18:1;O2/16:0, might be markers for an early stage of granuloma development. This stage is termed pregranulomatous exudative (PE) stage in the literature and an initial recruitment of leucocytes (T- and B-cells) is typical.^45^ A schematic cell model of this stage is shown in Figure S8a. After further granuloma development, the granulomatous exudative-productive (EP) stage is formed. The EP stage is characterized by a highly ordered structure with macrophages and eosinophils as the inner layer, surrounded by fibroblasts/hepatic stellate cells, and a collagen layer, which is surrounded by an outer layer of T- and B-cells (Figure S8b).^38^ Based on the infection time of the hamsters and the size of the granulomas, most were probably in the EP-stage. In these regions, we detected HexNac_2_Hex_3_Cers as the main granuloma markers, all with the same lateral distribution, similar to HexNac_2_Hex_3_Cer 18:1;O2/16:0 (Figure 2b, green). Because these GSL compounds were not observed during the assumed PE-stage, they were potentially formed during macrophage or eosinophil infiltration. In addition, we find for the suggested EP-stage granulomas a slight accumulation of HexNacHex_3_Cer 18:1;O2/16:0 in the outer layer of the granulomas, as shown in Figure S2i, which is in line with the model of the EP-stage. To further substantiate our assumption, AP-SMALDI MSI combined with IHC could help to link GSL distributions to specific cell types. A first example of IHC compared to ion images obtained by AP-SMALDI MSI is shown in Figure S9, where tissue sections neighboring those used for AP-SMALDI MSI were analyzed. While the highlighted granuloma in Figure S9 (top row) indicates the presence of HexNacHex_3_Cer 18:0;O2/16:0 throughout the granuloma and also T-cell-specific CD3-staining across the granuloma, the bottom row only shows signals at the outer layer of the granuloma for HexNacHex_3_Cer 18:0;O2/16:0 as well as CD3-positive cells. Due to the limited reactivity of available antibodies with hamster antigens, immunofluorescence experiments are challenging. Nonetheless, our experiments provide the first hints for a possible link between GSLs and infiltrating immune cells.

To semiquantitatively evaluate the increased signal intensities of specific GSLs in granuloma/egg compared to the surrounding tissue, signal intensities of each selected GSL were summed up in a defined ROI and normalized to the TIC of the ROI. For three GSL compounds, Fuc_3_HexNac_6_HexCer 20:0;O3/16:0, HexNac_2_Hex_3_Cer 18:1;O2/16:0, and HexNacHex_3_Cer 18:1;O2/16:0, the results are shown in Figure 2i. The normalized signal intensity per pixel of Fuc_3_HexNac_6_HexCer 20:0;O3/16:0 for granuloma and eggs of bs-infected samples was found to be 180- to 1400-fold increased compared to the other three ROI (normal tissue of bs-infected, ss-infected, and noninfected samples). For the compounds HexNac_2_Hex_3_Cer 18:1;O2/16:0 and HexNacHex_3_Cer 18:1;O2/16:0, the signal intensities per pixel were found to be 350- to 1100-fold and 8- to 28-fold increased, respectively. The semiquantitative data were also in line with our nano-HILIC MS/MS data, where we observed a significant increase (*p* < 0.001) for the compounds Fuc_3_HexNac_6_HexCer 20:0;O3/16:0, HexNac_2_Hex_3_Cer 18:1;O2/16:0, and HexNacHex_3_Cer 18:1;O2/16:0. These results for neutral GSLs indicated that specific GSL regulation in granuloma occurred upon *S. mansoni* infection, providing potential GSL markers for granuloma formation.

In total, we were able to visualize 21 neutral GSLs out of the 31 compounds identified via nano-HILIC MS/MS and to locally pinpoint most of these GSL species, significantly upregulated in bs-infected tissue, to granuloma/eggs. An earlier study of cancer tissue already demonstrated the analysis of neutral complex GSLs by high-resolution MSI with a 15 μm step size.^5^ With our AP-SMALDI MSI setup, we were able to routinely use a spatial resolution of 10 μm step size to resolve the different morphological structures in our samples. The high mass resolution and accuracy provided by our orbitrap mass spectrometer was essential for signals in the lower mass range (700–1000) to obtain true assignments and authentic distributions of compounds.

To characterize GSL regulation in granulomas, GSL species preferentially ionized in negative-ion mode are described in the next section.

### Acidic Glycosphingolipids Were Enriched in Distinct Areas of Hepatic Granuloma

**Figure 3:**
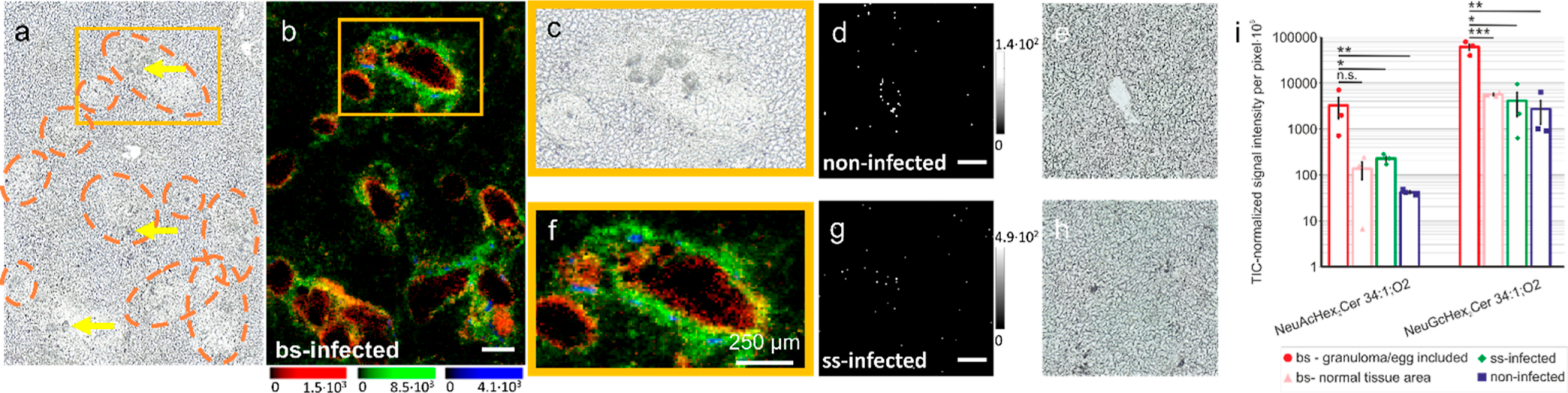
AP-SMALDI analysis of acidic GSLs. (a) Microscopic image of a liver tissue section of a bs-infected hamster, with yellow arrows exemplarily pointing at *S. mansoni* eggs and orange-dotted circles highlighting granuloma. (b) RGB image corresponding to the microscopic image in (a), showing NeuAcHex_2_Cer 18:1;O2/16:0 ([M–H]^−^ at *m*/*z* 1151.7058) in red, NeuGcHex_2_Cer 18:1;O2/16:0 ([M–H]^−^ at *m*/*z* 1167.7008) in green, and SHexCer 18:1;O2/16:0 ([M–H]^−^ at *m*/*z* 778.5148) in blue. Magnifications of (a,b) are shown in (c,f). (d) Ion image of a liver tissue section of a noninfected hamster of NeuGcHex_2_Cer 18:1;O2/16:0 ([M – H]^−^ at *m*/*z* 1167.6977) with the corresponding microscopic image (e). (g) Ion image of a liver tissue section of an ss-infected hamster of NeuGcHex_2_Cer 18:1;O2/16:0 ([M – H]^−^ at *m*/*z* 1167.6987) with corresponding the microscopic image (h). Scale bars indicate a length of 250 μm. (i) Histograms for the GSL species shown in the RGB-overlay based on the semiquantitative analysis of AP-SMALDI data. Black lines centered above two bars indicate the difference between the two corresponding ROIs, with “***” representing a significant difference with *p* < 0.001, “**” with *p* < 0.01, and “*” with *p* < 0.05. Error bars show the standard error.

AP-SMALDI MSI measurements were conducted in negative-ion mode for the detection of acidic GSLs. In total, ion images of 32 GSLs with five different saccharide compositions were generated for bs-infected samples. The acidic GSL species were found to contain NeuGcHex_2_, NeuAcHex_2_, NeuGc_2_Hex_2_, and NeuGcHexNacHex_2_. In addition, neutral GSLs were also visualized in negative-ion mode. As an example, HexNac_2_Hex_3_Cer 18:1;O2/16:0 is shown as the deprotonated species (Figure S10l). The negative-ion mode can thus be employed for cross validation of results obtained in the positive-ion mode. Representative AP-SMALDI images for acidic GSLs and corresponding microscopic images are shown in Figure 3. The spatial distributions for acidic GSLs showed specific accumulations within the granulomas. Here, acidic GSLs containing NeuGc and NeuAc were found to differ.

As an example, the compound NeuAcHex_2_Cer 18:1;O2/16:0 at *m*/*z* 1151.7058 is shown in red in Figure 3b. It exhibited high signal intensities in the middle layer of the granulomas, referring to the proposed granuloma model of the EP-stage (Figure S8b). In contrast, the compound NeuGcHex_2_Cer 18:1;O2/16:0 at *m*/*z* 1167.7009, shown in green (Figure 3b), exhibited high signal intensities in the outer layer of granulomas. The compound was also detected in the surrounding tissue of the granulomas. Therefore, this compound might correspond to T- and B-cells, which are the main cells of the outer granuloma layer. Small populations of these cells are also expected in healthy hepatic tissue. Additionally, in the RGB image (Figure S11b), the compound HexNac_2_Hex_3_Cer 18:0/16:0 is shown in red. This compound may represent a marker for the inner layer of the EP-stage. Together with the compounds NeuAcHex_2_Cer 18:1;O2/16:0 and NeuGcHex_2_Cer 18:1;O2/16:0, we were able to describe all three different layers of the granuloma in the EP-stage with different GSL compositions.

Moreover, we detected only five compounds containing a NeuAc moiety, all showing a similar spatial distribution. Species containing a NeuGc moiety were detected in various molecular compositions. For NeuGcHex_2_, 13 different ceramide compositions were detected, with examples in Figure S10a–d. In the noninfected sample shown in Figure 3d, NeuGcHex_2_Cer 18:1;O2/16:0 at *m*/*z* 1167.6977 was detected with low signal intensities around the blood vessels, whereas no accumulation was observed for the ss-infected sample (Figure 3g).

Another aspect becomes apparent when analyzing the data sets, which is the possibility of monitoring GSL metabolic processes. An example highlighting the benefits of combining nano-HILIC MS/MS and AP-SMALDI MSI data for metabolic GSL transformations during immune responses is the known conversion of NeuAc into NeuGc GSLs.^46^ In hamsters and other mammals, the enzyme cytidine monophosphate-*N*-acetylneuraminic acid hydroxylase (CMAH) catalyzes the required transformation. GSLs containing these head groups are shown in Figure 3b, namely, NeuGcHex_2_Cer 18:1;O2/16:0 in green and NeuAcHex_2_Cer 18:1;O2/16:0 in red. These compounds were found to locally overlap and to be significantly upregulated upon infection. Thus, it is tempting to speculate that CMAH activation during immune response results in increased production of NeuGcHex_2_Cer 18:1;O2/16:0. If this hypothesis is correct, the tools developed here could allow one to track specifically the end product of enzymatic cascades as a function of time and link the GSL expression profiles to specific regions with altered immune activity during *S. mansoni* infection. This could help to systematically investigate GSL transformations in tissues during disease progression. The advantage of AP-SMALDI MSI with the simultaneous visualization of various compounds outperforms classical, targeted imaging techniques.

In addition to the detectability of acidic GSL compounds, the negative-ion mode is also suitable for the detection of sulfated GSLs. As an example, SHexCer 18:1;O2/16:0 at *m*/*z* 778.5148 is shown in blue in Figure 3b, partially surrounding the granulomas. The same distribution pattern was observed for seven other SHex compounds with different ceramide compositions (Figure S10e–k). For sulfated compounds, our annotations were based on the accurate mass only because we did not detect these compounds with nano-HILIC MS/MS. Here, the advantage of the orthogonality of MALDI MSI and nano-HILIC MS/MS becomes apparent. While nano-HILIC MS/MS allows for a more detailed structural elucidation of GSL compounds, it does have some limitations. If, for example, species like SHexCers are only partially accumulated throughout the whole tissue, then they will be possibly too low in concentration in a bulk analysis. In contrast, by using MALDI MSI and therefore maintaining spatial information, high local concentrations make the detection and visualization of such species possible.

The semiquantitative evaluation of NeuAcHex_2_Cer 18:1;O2/16:0 and NeuGcHex_2_Cer 18:1;O2/16:0 signals is presented in Figure 3i. For the two compounds, the TIC-normalized signal intensities per pixel were found to be increased 14- to 78-fold and 11- to 22-fold, respectively, for granuloma/egg ROIs compared to those for the other three ROI (normal tissue of bs-infected samples and ss- and noninfected samples). These results are in line with our nano-HILIC MS/MS data, where significant differences in these GSLs were observed between bs-infected samples and controls. Out of the 30 GSLs identified by nano-HILIC, negative-ion mode AP-SMALDI MSI allowed local tracking of the distribution of 17 of these compounds. These acidic GSLs were predominantly accumulated around or on the outer borders of granulomas. In addition, these results are consistent with the evaluation of positively charged GSLs. Together with the positive-ion mode analysis, a comprehensive and locally resolved overview of GSLs involved in immune response during *S. mansoni* infection and granuloma formation was obtained. As reported in the literature and confirmed by our initial experiments, DHAP is the matrix of choice for AP-SMALDI measurements of acidic GSL.^42,43^ Whereas most studies focused on brain tissue,^47−49^ we show the first application to hamster liver tissue. We also demonstrate that high-resolution AP-SMALDI MSI of GSLs can be routinely performed with a 10 μm step size. Importantly, we included no additional washing steps for the tissue sections prior to analysis. This is relevant when comparing our study to the literature, where at a lower spatial resolution, an increased detection sensitivity of acidic GSL was achieved by additionally washing the tissue slide.^48^ While additional washing steps can be useful to increase sensitivity, they generally limit the spatial resolution due to wash-out effects.

### Mass Spectrometry Imaging Was Optimized Down to 3 μm Lateral Resolution

**Figure 4:**
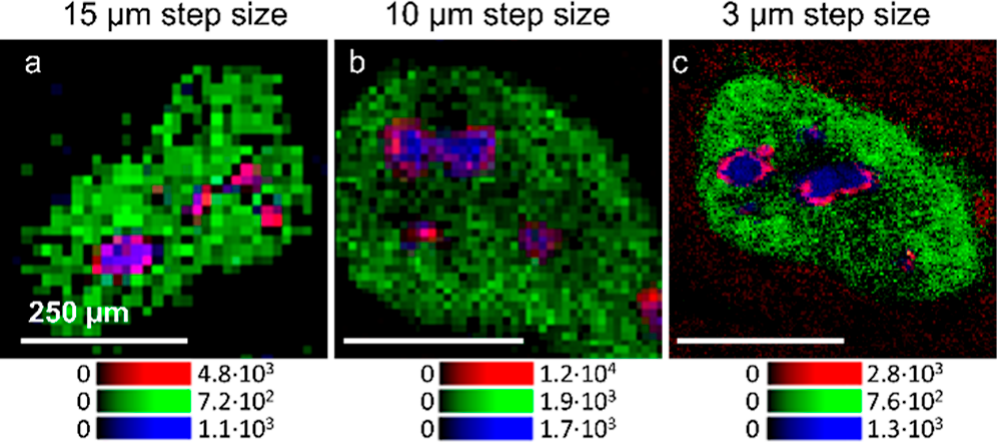
Increasing the lateral resolution enables the localization of substructures in *S. mansoni* eggs. (a) RGB overlay images of three granulomas measured with a 15 μm step size, (b) 10 μm step size, and (c) 3 μm step size using an experimental AP-SMALDI imaging setup, showing HexCer 20:0;O3/16:0 ([M + K]^+^ at *m*/*z* 784.5715) in red, HexNac_2_Hex_3_Cer 18:1;O2/16:0 ([M + K]^+^, at *m*/*z* 1468.7913) in green, and PC 38:1 ([M + K]^+^ at *m*/*z* 854.6042) in blue.

In order to distinguish additional histological features within granulomas at the molecular level, the effective lateral resolution of the method was further improved by employing an experimental ion source setup with a smaller laser focus. Mass spectrometric images with 15, 10, and 3 μm pixel size are shown for comparison in Figure 4. The RGB overlay images show HexCer 20:0;O3/16:0 in red, HexNac_2_Hex_3_Cer 18:1;O2/16:0 as a granuloma marker in green, and PC 38:1 as a marker for *Schistosoma* eggs in blue. HexCer 20:0;O3/16:0 can be assigned to the surface of the *S. mansoni* eggs from the 10 μm step size image but not from the 15 μm step size image. The assignment and interpretation are obviously much clearer and more trustworthy when using the 3 μm experimental setup and method (Figure 4c). These first experiments, using DHAP as a matrix, demonstrate the capability of our workflow to track GSLs with cellular resolution.

## Conclusions

In this study, we optimized a high-resolution AP-SMALDI MSI approach in combination with nano-HILIC MS/MS to reveal the global and local hepatic GSL profiles of liver samples of *S. mansoni*-infected hamsters compared to that of noninfected controls. Overall, we found statistically significant differences between the hepatic GSL profiles of infected and noninfected animals. Based on high-resolution AP-SMALDI MSI data, upregulated GSLs primarily localized within granulomas surrounding *S. mansoni* eggs in the liver. This suggests that GSL topology is associated with granuloma formation, potentially related to infiltrating and differentiating immune cells. Whether the determined GSL species are merely markers for granuloma formation and whether they are associated with particular cell states and used for intercellular communication or cell differentiation will be a matter of future longitudinal studies. Linking GSL topography with specific granuloma stages is crucial to reveal the time courses of GSL development and to connect GSL species with defined immune cell differentiation stages.

## Supporting Information Available

Additional experimental protocols, experimental parameters, data-processing steps, additional results for the matrix evaluation of DHAP compared to other matrices, sublimation setup, more ion images for neutral GSL of bs-infected hamsters, structures of a GSL and monosaccharide units, MS^2^ spectra of HexNac_2_Hex_3_Cer 18:1;O2/16:0 in negative-ion mode, H&E-stained section and the corresponding RGB-overlay of the liver of a bs-infected hamster, granuloma model for the PE- and EP-stages, IHC results compared to MALDI MSI results, and additional ion images for acidic GSL and an H&E-stained section and the corresponding RGB-overlay of the liver of bs-infected hamsters with three different distributions of GSLs within a granuloma (PDF)
Glycosphingolipid database (XLSX)
