# Supplementary material for: Hepatic Topology of Glycosphingolipids in Schistosoma mansoni-Infected Hamsters
Source: Anal Chem. 2024 Apr 9;96(16):6311–20. doi: 10.1021/acs.analchem.3c05846 (PMC11044111; doi:10.1021/acs.analchem.3c05846)
Supplement: Supplementary file 1 — ac3c05846_si_001.pdf [file ac3c05846_si_001.pdf]

Supporting information

Hepatic topology of glycosphingolipids in *Schistosoma mansoni*-infected hamsters

David Luh[1], Sven Heiles[1,2,3], Martin Roderfeld[4], Christoph G. Grevelding[5], Elke Roeb[4] and Bernhard Spengler[1,*]

[1]Institute of Inorganic and Analytical Chemistry, Justus Liebig University Giessen, 35392 Giessen, Germany

[2]Leibniz-Institut für Analytische Wissenschaften - ISAS - e.V., 44139 Dortmund, Germany

[3]Lipidomics, Faculty of Chemistry, University of Duisburg-Essen, 45141 Essen, Germany

[4]Gastroenterology, Justus Liebig University Giessen, 35392 Giessen, Germany

[5]Institute for Parasitology, Justus Liebig University Giessen, 35392 Giessen, Germany

Table of contents

Supplementary Protocol S1	S3
Supplementary Protocol S2	S3
Supplementary Note S1	S4
Supplementary Note S2	S4
Supplementary Note S3	S4
Supplementary Note S4	S4
Supplementary Note S5	S5
Supplementary Table S1	S6
Supplementary Table S2	S7
Supplementary Table S3	S7
Supplementary Table S4	S8
Figure S1	S8
Figure S2	S9
Figure S3	S10
Figure S4	S11
Figure S5	S12
Figure S6	S13
Figure S7	S13
Figure S8	S14
Figure S9	S15
Figure S10	S16
Figure S11	S17

Supplementary Protocol 1: Sample preparation for nano-HILIC MS/MS measurements

For nano-HILIC MS/MS, GSLs were extracted as follows. Starting with hamster liver homogenate, 240 µL water, 640 µL methanol and 320 µL chloroform were added, followed by a two-hour incubation at 38 °C with intermediate mixing every 15 minutes. The suspension was centrifuged with 1400 g for 5 minutes. The supernatant was removed and kept on ice. With the remaining pellet, the previous steps were repeated and the supernatants were combined. Solvents were evaporated under a stream of nitrogen. Subsequently, the samples were dissolved in 1.5 mL of 0.1 mol/L sodium hydroxide in methanol and incubated at 38 °C for saponification of phospholipids. This was followed by neutralization of the sample solutions with glacial acetic acid and evaporation of the solvents under a nitrogen flow.

For purification and desalting, dried GSLs extracts were dissolved in chloroform/methanol/water (3:98:74, v/v/v) and applied to a C18-SPE cartridge (Chromabond C18ec, Macherey and Nagel, Düren, Germany), according to Wuhrer et al.[1] Before applying the sample, the cartridges were washed with 10 column volumes of methanol, 10 column volumes of chloroform/methanol (2:1, v/v) and again 10 column volumes of methanol. Equilibration was done with five column volumes of chloroform/methanol/water (3:98:74, v/v/v). After applying the sample, the SPE cartridges were washed with 10 column volumes of water and the sample was then eluted with five column volumes of methanol. The samples were dried under a stream of nitrogen, dissolved in 1.5 mL methanol/water (1:1, v/v) and further diluted by a factor of 15 with the same solvents for analysis. The pooled quality control sample was a mix of 1 µL from the stock solution of each second biological replicate with in total 91 µL methanol/water (1:1, v/v).

Supplementary Protocol 2: H&E staining protocol

Hematoxylin and eosin (H&E) staining was performed after AP-SMALDI MSI measurements. First, matrix was rinsed off with ethanol and then dehydrated for 2 min in ethanol followed by a 2 min incubation in 70% ethanol. Then the sample was incubated for 2 min in 40% ethanol followed by a 2 min incubation step in HPLC-grade water. Staining with hematoxylin for 12 min, washing with tap water for 15 min and staining with 1% Eosin Y solution for 1 min and differentiation with HPLC-grade water for 2 min, 40% ethanol for 2 min, 70% ethanol for 2 min and 100% ethanol for 2 min were subsequently carried out. Clearing with xylol for 2 min and covering with Eukitt and a cover slide completed the staining protocol.

Supplementary Note 1: Experimental parameters for AP-SMALDI analysis

For AP-SMALDI MSI measurements, the mass range was set to *m/z* 700-2,800 for 10 µm and 15 µm step size measurements and *m/z* 600-1,800 for 3 µm step size measurements, respectively. Measurements were performed in 2-D pixel mode[2] with 50 laser pulses per pixel at a wavelength of 343 nm. Capillary temperature was set to 250 °C and the S-lens was set to 100. For Q Exactive HF, the acceleration voltage was set to ±3 kV and for Q Exactive, the acceleration voltage was set to 2.5 kV.

Supplementary Note 2: Processing for statistical evaluation of AP-SMALDI MSI data

For statistical comparison the mean intensities per pixel were loaded into Perseus for each biological replicate (n=3). After a $\log_2$ transformation, a two-sided student's T-test was performed, were the ROIs of tissue sections including granulomas/eggs of bs-infected hamsters was compared to each of the remaining three groups (ROIs of liver tissue sections without granulomas/eggs of bs-infected hamsters, ROIs of liver tissue sections of ss- and non-infected hamsters).

Supplementary Note 3: Experimental parameters for HILIC MS/MS analysis

In short, solvent A consisted of acetonitrile/methanol (99/1, v/v) and solvent B of methanol/water (8/2, v/v), both with 5 mmol/L ammonium acetate. For injection, the pre-concentration setup was used with microliter-pickup injection. The sample injection volume was 1 µL. Further parameters are described in detail in the Table S2/S3.

Supplementary Note 4: Data Processing – nano-HILIC

For relative quantification of GSL species we chose SM 40:1;O2 as a endogenous reference, as no significant difference was observed between the three sample types in the nano-HILIC MS/MS data, where experiments with three technical replicates for each biological replicate (n=3) were conducted. As can be seen from the ion-image of SM 40:1;O2 shown in Figure S3 b, the signal was homogeneously distributed over the tissue but no signal was observed for the eggs. The .raw files were converted with msconvert to mzXML-files, which were uploaded in MZMine 2.33 and GSLs were identified based on our manually curated database. The coefficient of variation (CV) for each identified GSL was calculated for the pooled QC sample, which was measured five times as a technical replicate. The peak areas were used for the calculation of the CV. The resulting box plot for positive- and negative-ion mode data is shown in Figure S4. While a median close to 20% for the CV values for the positive-ion data indicates a sufficient reproducibility, a median close to 50% for the CV in the negative ion-mode should be optimized in further studies. Therefore, we decided to perform our relative quantification based on our positive-ion mode data. Here, the initial PCA plot including all biological and

technical replicates is shown in Figure S3 c. Based on optical evaluation, bs77_1, highlighted in the PCA plot, was excluded from further statistical analysis. The peak areas were used for relative quantification to the endogenous standard and mean and standard error were calculated in Excel. Data was uploaded in Perseus and the samples were categorially annotated into bs-, ss- and non-infected samples types. ANOVA tests with p-values of 0.05, 0.01 and 0.001 were used and significant different groups identified by a post hoc tests with an FDR of 0.05. Although no exogenous standards were utilized in this study, we believe that normalization to the endogenous species SM 40:1, which showed comparable peak areas throughout all sample types, provides a semi-quantitative statement. However, the use of exogenous internal standards would increase the precision of the semi-quantitative statements and would allow to move towards absolute quantification. Unfortunately, availability of deuterated GSL standards is very limited.

Supplementary Note 5: DHAP compared to DHB, CHCA, 9-AA and DAN

When comparing matrices regarding the number of annotations in positive-ion mode using LIPIDMAPS, we found 111 annotations for phospholipids and 1 triglyceride with DHAP matrix. For DHB and CHCA matrix, the number of annotations were 82 and 55 for phospholipids, and 0 and 1 for triglycerides, respectively. For the negative-ion mode, again more lipids were annotated with DHAP matrix (30 phospholipids, 11 SHex compounds) than with 9-AA (26 phospholipids, 11 SHex compounds) and DAN matrix (28 phospholipids, 10 SHex compounds).

Supplementary Table 1. Protocols for matrix application, sublimation parameters for DHAP and pneumatic spraying parameters for used matrices

Matrix	Parameters
DHAP	- oil bath 130 °C<br>- cool finger -15 °C to -20 °C<br>- duration 5 minutes
9-AA	-7 mg/mL<br>-acetone/water 6/4<br>-5 µL/min<br>-160 µL
DAN	- 3 mg/mL<br>- methanol/water; 9/1; v/v<br>-15 µL/min<br>- 225 µL
DHB	- 30 mg/mL<br>- acetone/water; 1/1; v/v + 0.1% TFA<br>- 10 µL/min<br>- 100 µL
CHCA	- 7 mg/mL<br>- acetone/water; 1/1; v/v + 0.1% TFA<br>- 10 µL/min<br>- 110 µL

Supplementary Table 2: nano-HILIC Parameter used for separation of glycosphingolipids.

Property	Setting
Mobile Phase A	acetonitrile/methanol (99/1, v/v), 5 mM AmAc
Mobile Phase B	methanol/water (8/2, v/v), 5 mM AmAc
Loading Solvents	same as starting conditions
Injection volume	1 µL
Loading Time	0.5 minutes
Gradient	5% B for 5 minutes, to 100% B in 45 minutes, hold 100% B for 10 minutes, to 5% B in 5 minutes, hold 5% B for 55 minutes
Oven temperature	40 °C
Autosampler temperature	4 °C
Loading flow rate	30 µL/min
NC flow rate	300 nL/min

Supplementary Table 3: MS and MS/MS parameters (in brackets) used for identification of GSL after nano-HILIC separation

Parameter	Setting
Spray voltage	±1.9 kV
Capillary temperature	350 °C
S-lens setting	100
Mass resolution at m/z 200	120000 (45000)
MS AGC target	1000000 (100000)
MS max. injection time	100 ms (50 ms)
Scan range	*m/z* 600 - 2400
Top N	15
Isolation window Δ (*m/z*)	0.8
Stepped NCE	20;25;30
Dynamic exclusion	3.6 seconds

Supplementary Table 4: Glycosphingolipid species with mean intensities per pixel for each matrix. DHAP was evaluated in positive- and negative-ion mode. The other matrices were either evaluated in positive- (DHB, CHCA), or negative-ion mode (9-AA, DAN).

Species	DHAP (+)	DHB	CHCA	DHAP (-)	9-AA	DAN
**HexCer 18:1;O2/18:0**	$4.56 \cdot 10^2$	$3.44 \cdot 10^2$	$1.86 \cdot 10^2$			
**HexCer 18:1;O2/20:0**	$1.11 \cdot 10^2$	$7.69 \cdot 10^1$	$3.29 \cdot 10^1$			
**HexCer 18:1;O2/22:0**	$7.73 \cdot 10^2$	$6.46 \cdot 10^2$	$3.34 \cdot 10^2$			
**HexCer 18:1;O2/24:1**	$4.81 \cdot 10^3$	$4.01 \cdot 10^3$	$2.27 \cdot 10^3$			
**GM1 18:1;O2/18:0**				$7.44 \cdot 10^2$	$3.61 \cdot 10^2$	$1.90 \cdot 10^2$
**GM2 18:1;O2/18:0**				$8.19 \cdot 10^1$	$3.58 \cdot 10^1$	$1.24 \cdot 10^1$

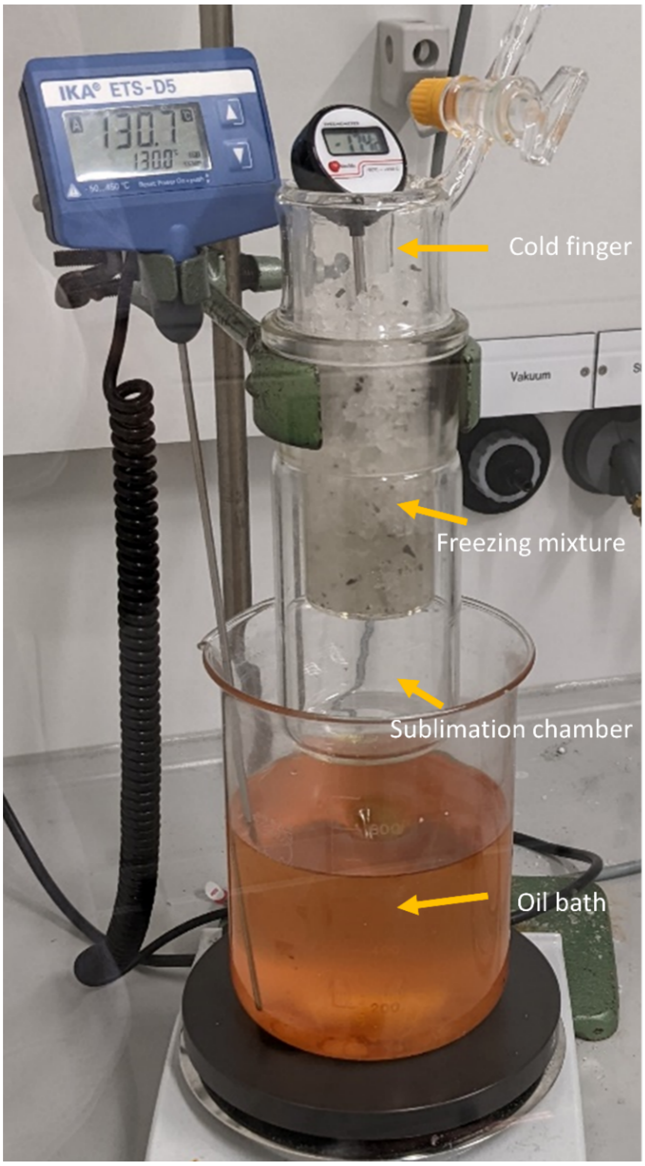

Figure S1: Sublimation apparatus. Arrows indicate the different components, namely the cold finger, the freezing mixture consisting of ice and salt, the sublimation chamber and the heated oil bath.

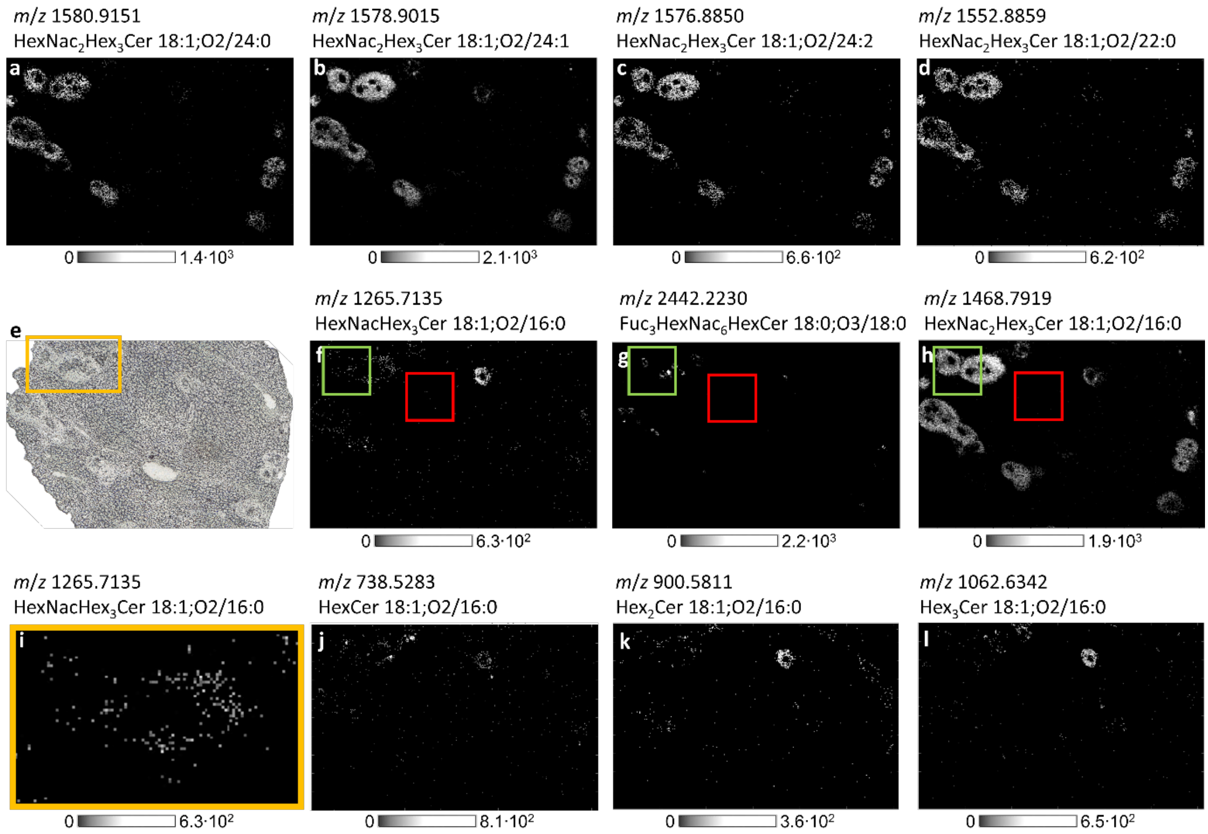

Figure S2: Various $HexNac_2Hex_3$ species with different ceramide compositions (top row + h), all representing the same spatial distribution as markers for most granulomas when compared to the corresponding microscopic image (e). Single-ion images (f-h) used for the RGB-overlay in Figure 2, showing $HexNacHex_3Cer$ 18:1;O2/16:0 at *m*/*z* 1265.7135 (f), $Fuc_3HexNac_6HexCer$ 20:0;O3/16:0 at *m*/*z* 2442.2230 (g), and $HexNac_2Hex_3Cer$ 18:1;O2/16:0 at *m*/*z* 1468.7919 (h). The green area represents the ROI used for statistical evaluation of the liver of a bisex-infected hamster including morphologically changed tissue upon infection compared to healthy hepatic tissue. The red area represents the ROI of the bisex-infected sample without granulomas or eggs used as a control for semi-quantitative analysis. Magnification of the single-ion image representing the distribution of $HexNacHex_3Cer$ 18:1;O2/16:0 at *m*/*z* 1265.7135 showing the granuloma area (i) highlighted by the orange square in the corresponding microscopic image. Ion images of HexCer 18:1;O2/16:0 at *m*/*z* 738.5283 (j), Hex_2Cer 18:1;O2/16:0 at *m*/*z* 900.5811 (k) and Hex_3Cer 18:1;O2/16:0 at *m*/*z* 1062.6342 (l), all showing a similar distribution as $HexNacHex_3Cer$ 18:1;O2/16:0 at *m*/*z* 1265.7135 (f). All ion images are created from the potassium-adduct signal.

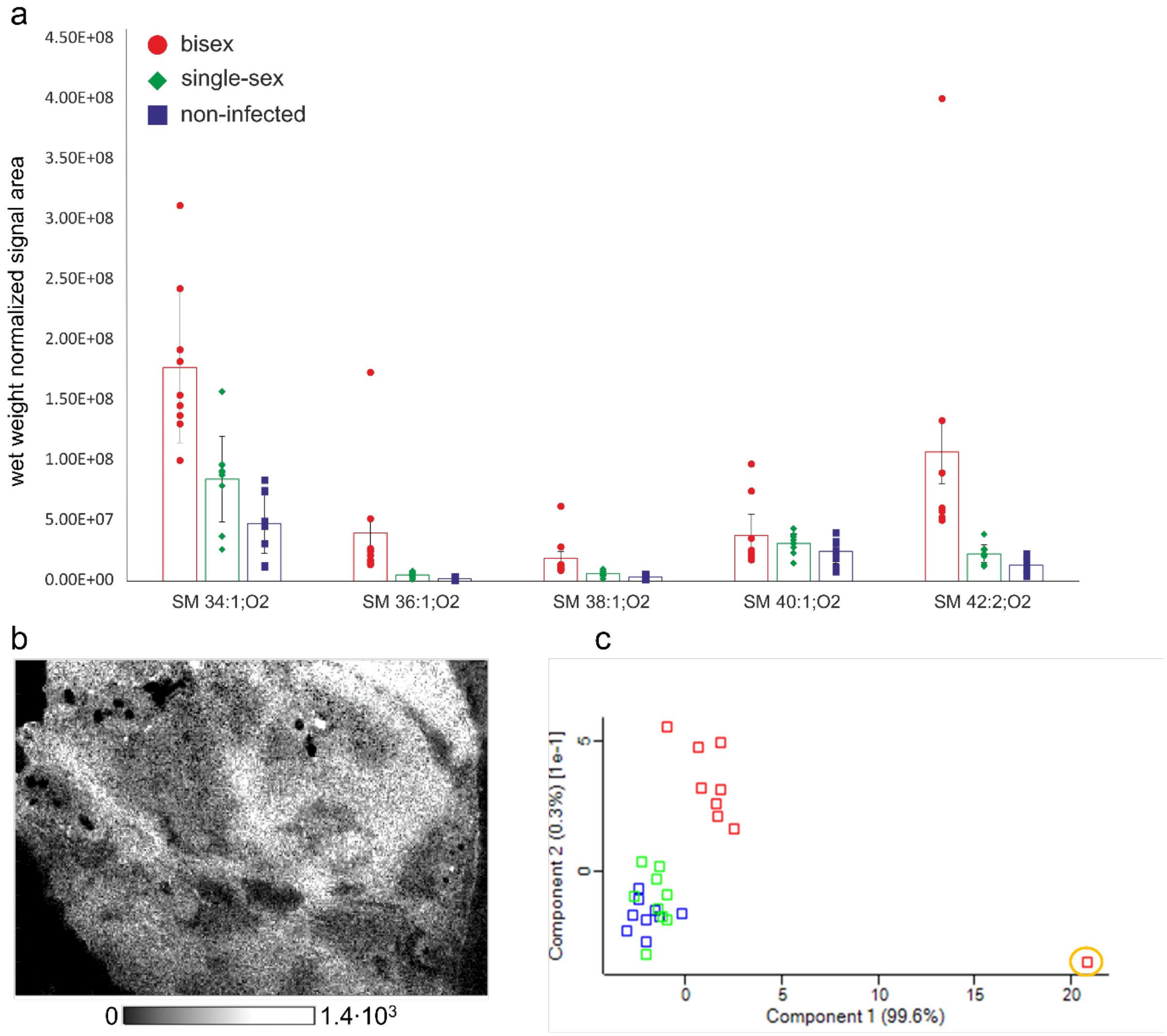

Figure S3: (a) Histograms for endogenous SM compounds showing the peak areas. Error bars indicate the standard deviation. (b) Representative ion-image of SM 40:1;O2 of a liver tissue section of a bs-infected hamster, indicating an almost homogenous distribution across the section with no signal for the eggs. (c) PCA plot including all three biological replicates with three technical replicates each for non-infected (blue), ss-infected (green) and bs-infected (red) GSL extracts based on nano-HILIC MS/MS data. The data point for bs77_1, highlighted by an orange circle, was not included in further statistical evaluation.

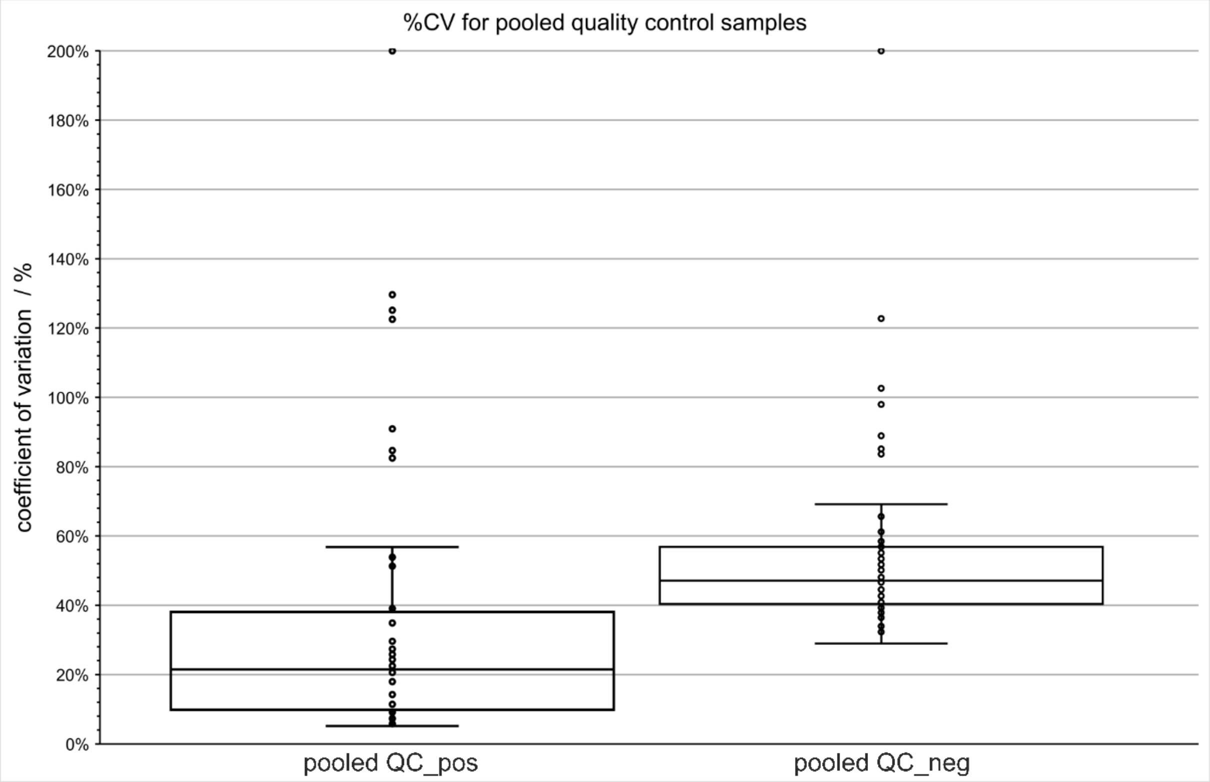

Figure S4: Box plot showing the coefficient of variation (CV). The %CV was calculated for each identified GSL species in positive- as well as negative-ion mode for the pooled QC sample, which was measured as a replicate five-times for each polarity.

I

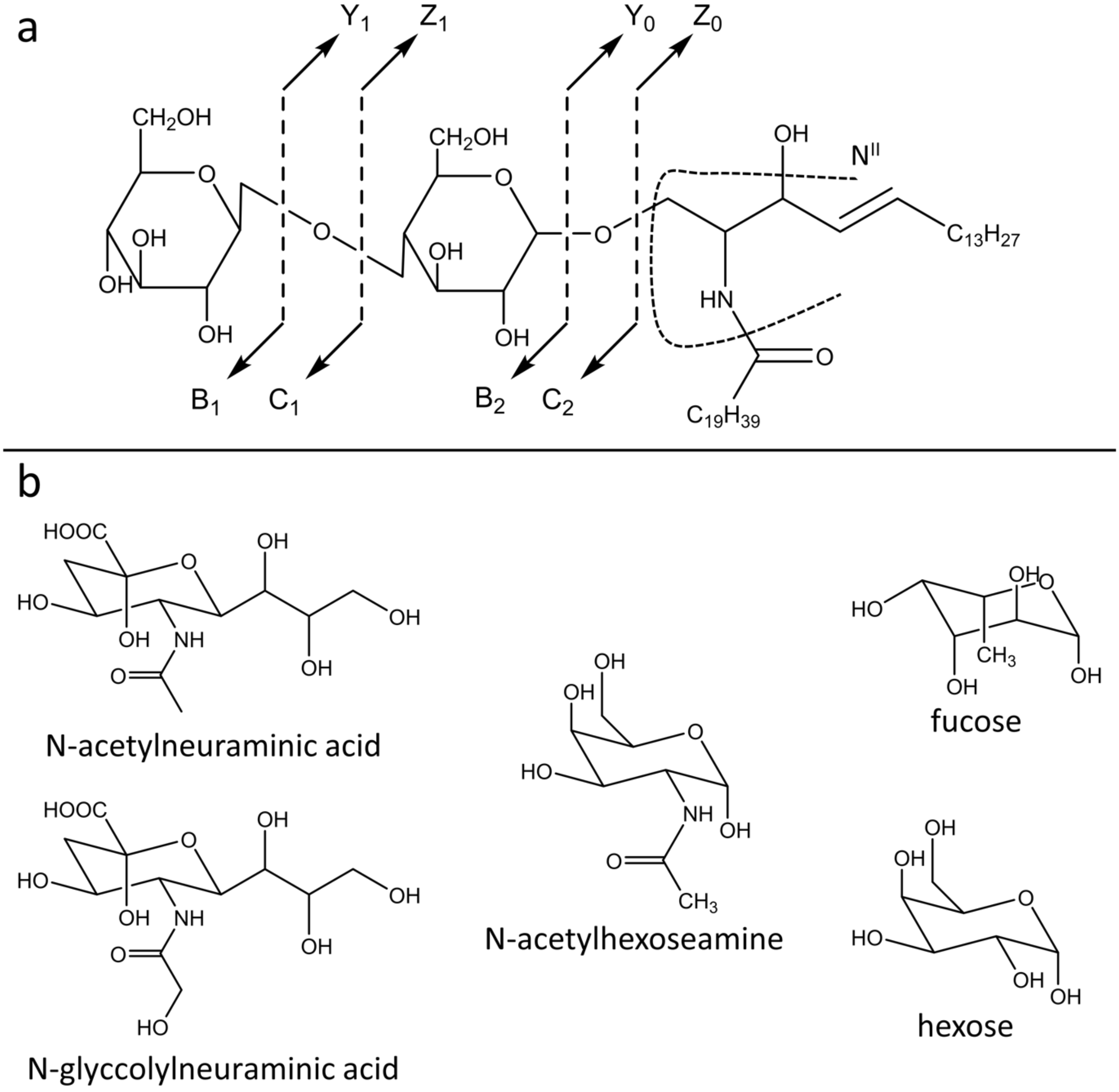

Figure S5: A GSL consisting of two monosaccharide units and a ceramide head group (a). Cleavage sites upon HCD fragmentation are indicated by dotted lines resulting in Y-, Z-, B, and C-fragment ions for the glyosidic cleavage of the saccharide units and the N^{II}-fragment ion for the sphingoid base. Nomenclature according to Domon and Costello[3] and Merrill et al.[4]. Different monosaccharide units, which can be discriminated by tandem mass spectrometry (b) showing NeuAc, NeuGc, HexNac, Fuc and Hex as the saccharides reported in this article.

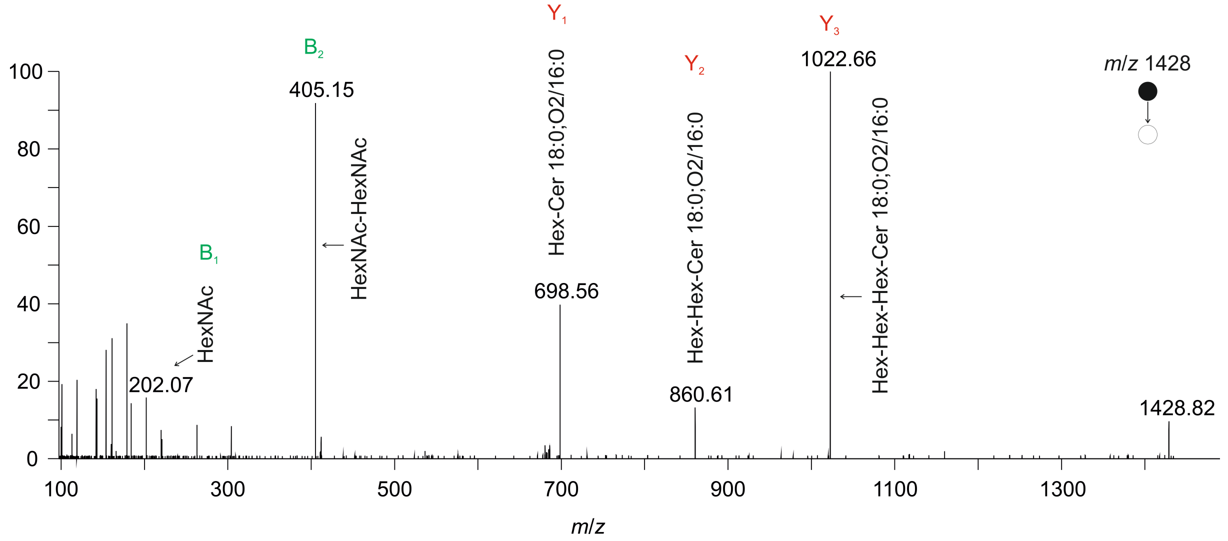

Figure S6: Tandem MS of [HexNac$_2$Hex$_3$Cer 18:1;O2/16:0 – H]$^-$ with specific fragment ions. B_1- and B_2-fragments for glyosidic cleavage by HCD beginning at the saccharide head-group, and Y_1-, Y_2- and Y_3-fragments for glyosidic cleavage by HCD counting from the ceramide-backbone are indicated. Fragment ion nomenclature according to Domon and Costello.[3]

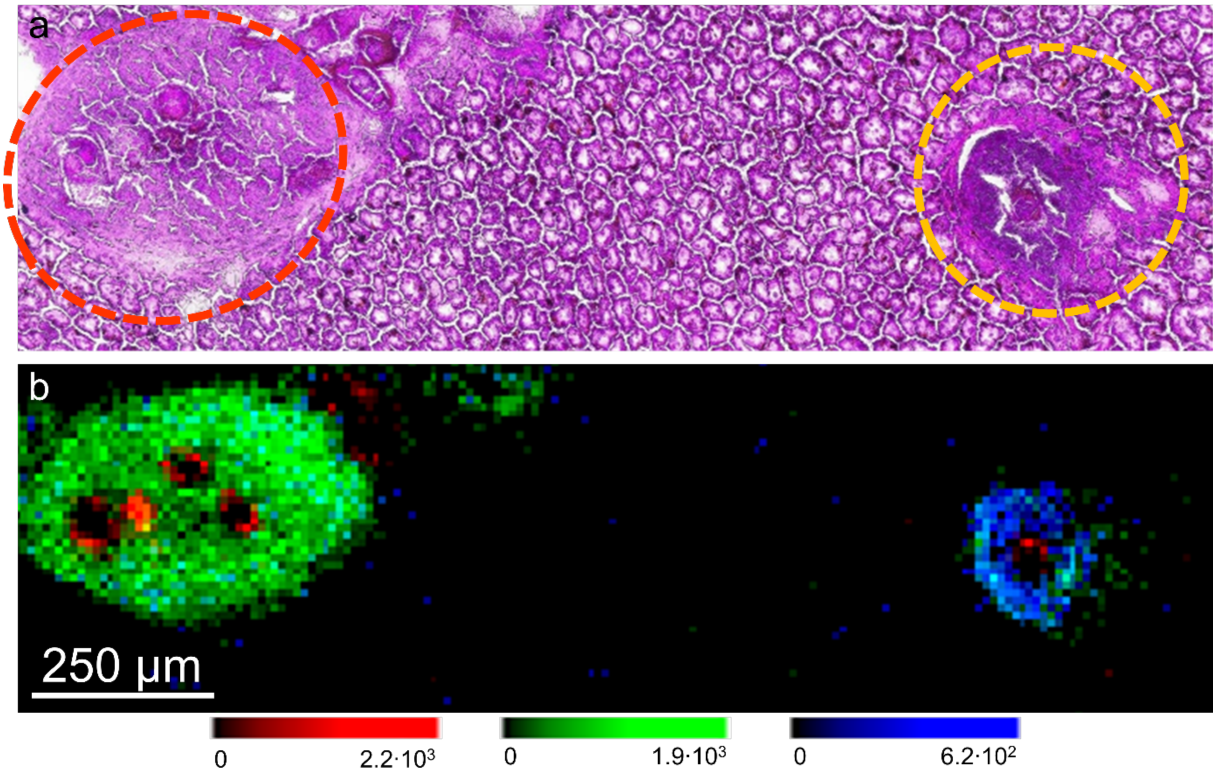

Figure S7: H&E-staining (a) of the tissue after AP-SMALDI MSI measurement showing two granulomas, one highlighted with a red-dotted circle and the other with an orange-dotted circle, respectively. Corresponding RGB image (b), showing Fuc$_3$HexNac$_6$HexCer 20:0;O3/16:0, [M+K]$^+$, at *m/z* 2442.2230 in red, HexNac$_2$Hex$_3$Cer 18:1;O2/16:0, [M+K]$^+$, at *m/z* 1468.7919 in green and HexNacHex$_3$Cer 18:1;O2/16:0, [M+K]$^+$, at *m/z* 1265.7135 in blue.

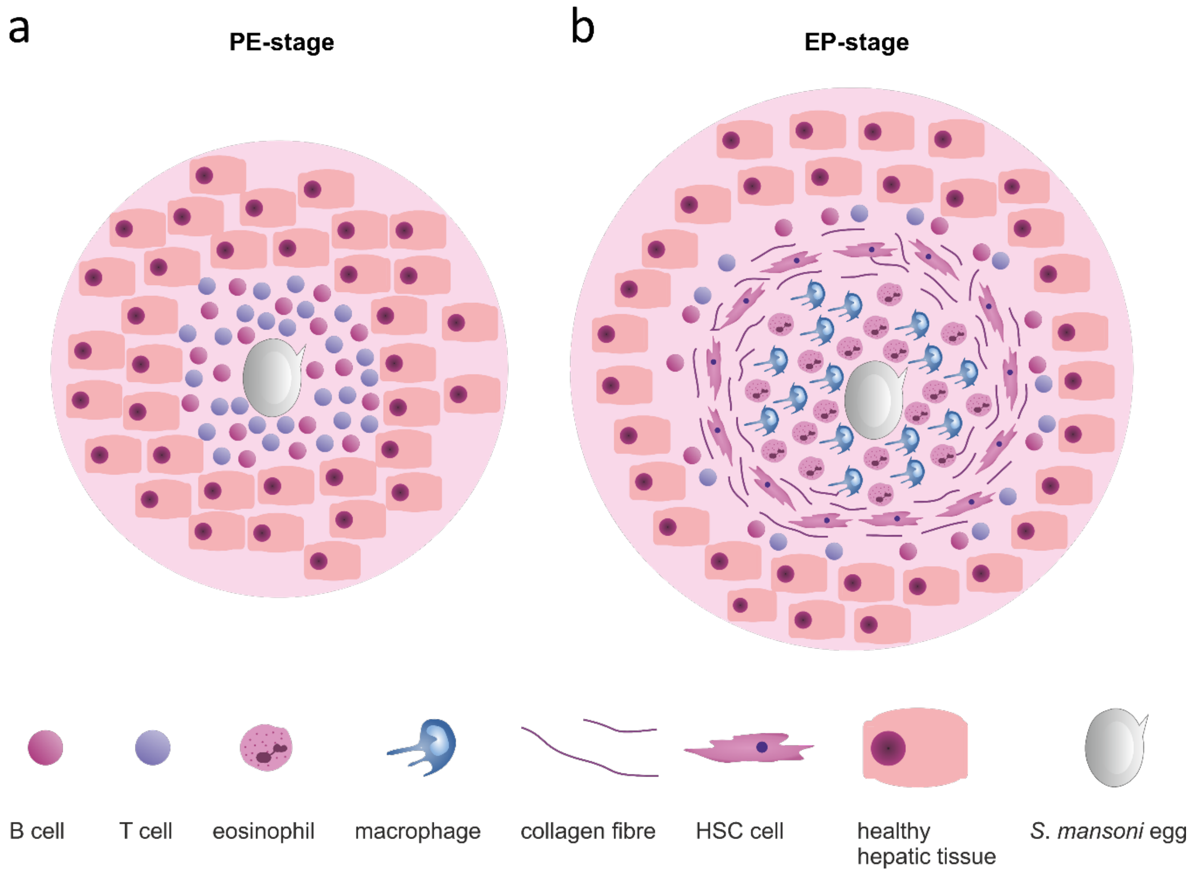

Figure S8: (a) Granuloma model of the pre-granulomatous exudative (PE) stage (a) with B and T cells surrounding the *S. mansoni* eggs. (b) Granuloma model of the exudative-productive (EP) stage, representing a highly ordered structure with eosinophils and macrophages as the inner layer around the egg, surrounded by collagen fiber and hepatic stellate cells as a middle layer and with B and T cells as the outer layer. In both granuloma stages, cell types with low abundance are not included in the model.

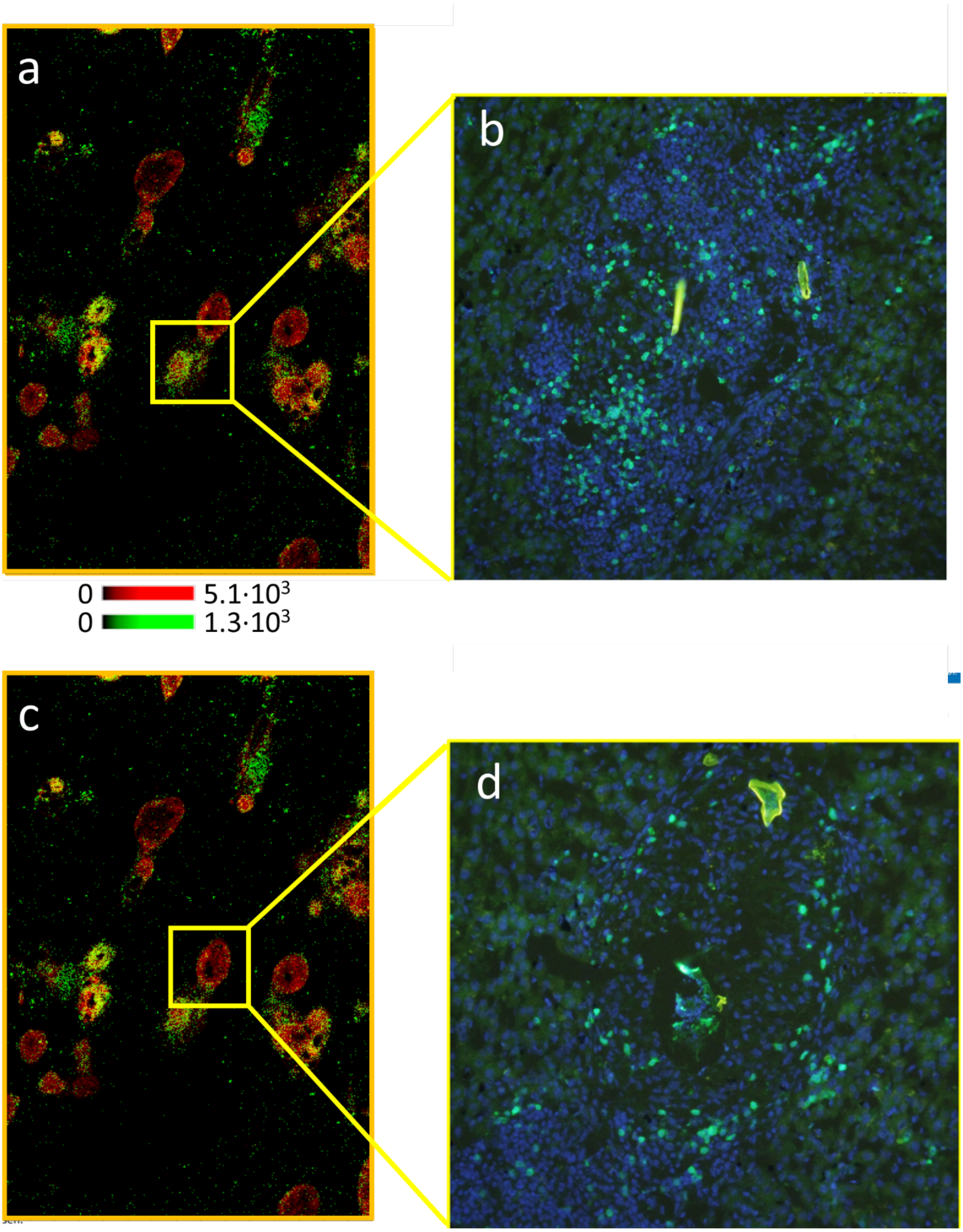

Figure S9: Red-green overlay images showing $HexNac_2Hex_3Cer$ 18:0,O2/16:0 at *m/z* 1468.7925 as $[M+K]^+$ in red and $HexNacHex_3Cer$ 18:0,O2/16:0 at *m/z* 1265.7131 as $[M+K]^+$ in green (a,c same section). Corresponding immunohistochemistry experiments with CD3 staining (green) and DAPI (blue) of the neighboring section of the AP-SMALDI measurement (b,d).

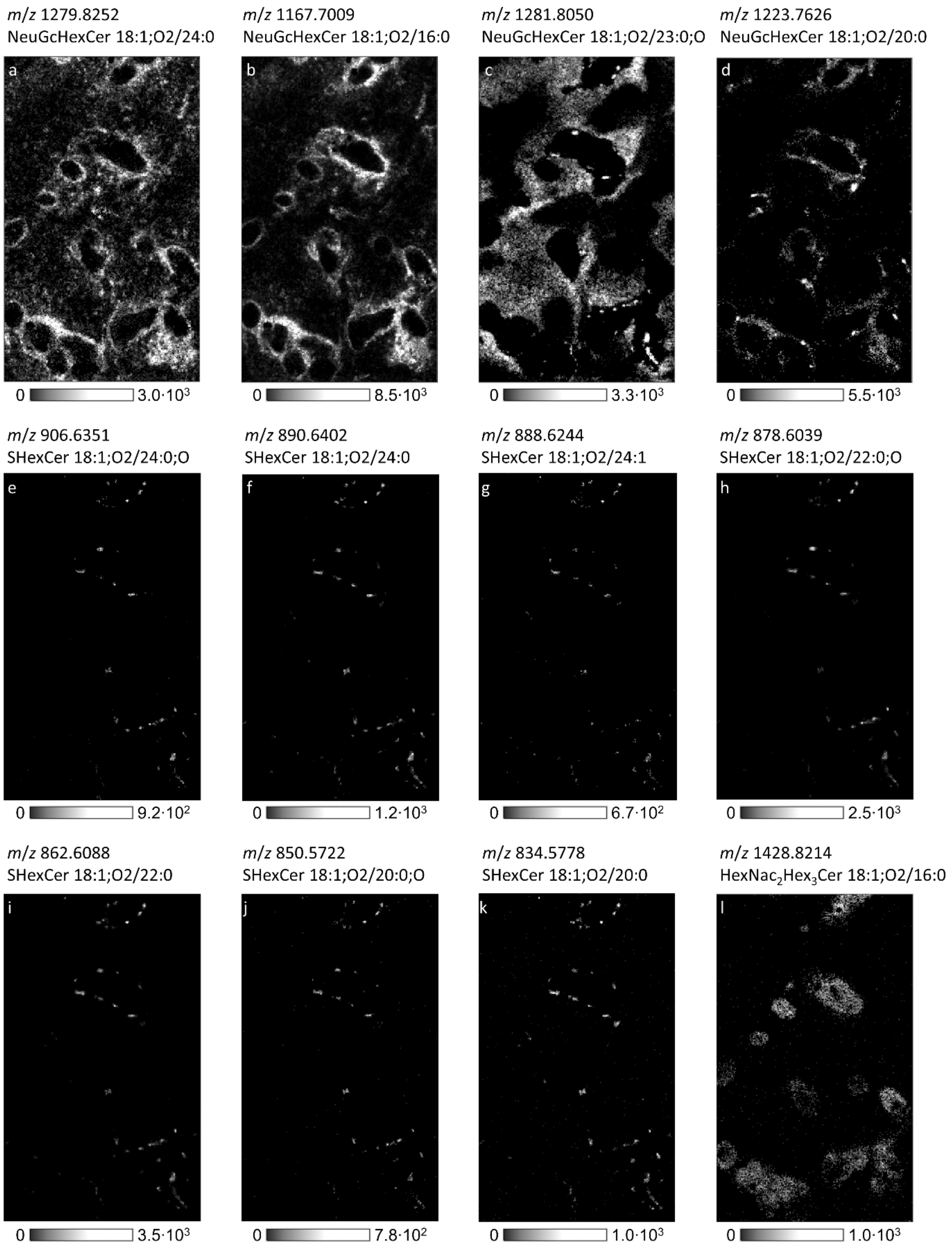

Figure S10: AP-SMALDI MS images of GSLs obtained in negative-ion mode as $[M-H]^-$. (a-d) NeuGcHexCer compounds with different ceramide compositions, showing distinguishable distributions. (e-k) SulfoHex compounds with different ceramides backbones, all showing similar distributions with accumulations at the outer surface of granulomas. (l) Neutral GSL $HexNac_2Hex_3Cer$ 18:1;O2/16:0. The corresponding microscopic image is shown in Figure 3a.

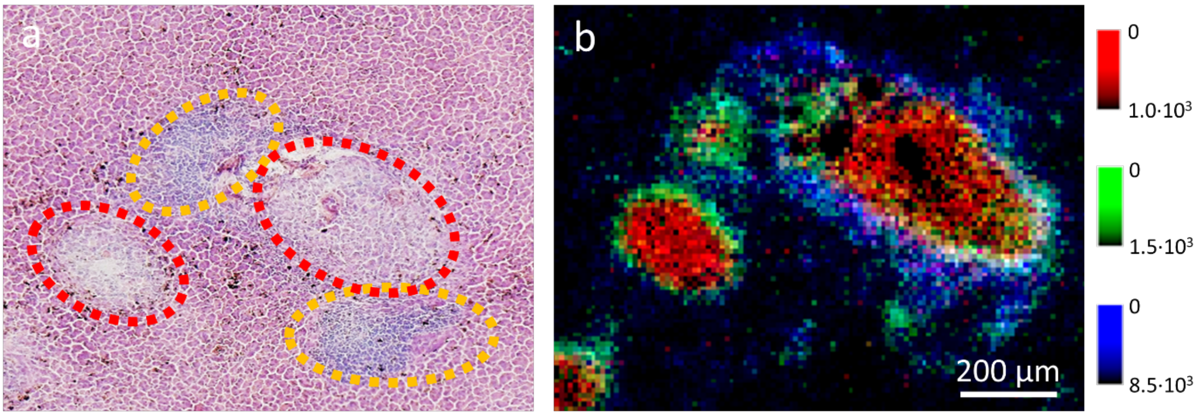

Figure S11. (a) H&E-stained liver tissue section of a bs-infected hamster after AP-SMALDI measurement. Granulomas are highlighted with red-dotted circles. Assumed accumulation of immune cells is highlighted with orange-dotted circles. (b) Corresponding RGB overlay image with $NeuGcHex_2Cer$ 18:0;O2/20:0 in blue, $NeuAcHex_2Cer$ 18:0;O2/16:0 in green and $HexNac_2Hex_3Cer$ 18:0;O2/16:0 in red.

References

(1) Meyer, S.; van Liempt, E.; Imberty, A.; van Kooyk, Y.; Geyer, H.; Geyer, R.; van Die, I. DC-SIGN Mediates Binding of Dendritic Cells to Authentic Pseudo-LewisY Glycolipids of Schistosoma mansoni Cercariae, the First Parasite-specific Ligand of DC-SIGN, *J. Biol. Chem.* 2005, 280, 37349-37359.

(2) Müller, M. A.; Kompauer, M.; Strupat, K.; Heiles, S.; Spengler, B. Implementation of a High-Repetition-Rate Laser in an AP-SMALDI MSI System for Enhanced Measurement Performance, *J. Am. Soc. Mass Spectrom.* 2021, 32, 465-472.

(3) Domon, B.; Costello, C. E. A systematic nomenclature for carbohydrate fragmentations in FAB-MS/MS spectra of glycoconjugates, *Glycoconjugate J.* 1988, 5, 397-409.

(4) Merrill, A. H.; Sullards, M. C.; Allegood, J. C.; Kelly, S.; Wang, E. Sphingolipidomics: high-throughput, structure-specific, and quanti-tative analysis of sphingolipids by liquid chromatography tandem mass spectrometry, *Methods* 2005, 36, 207-224.
